# Generation of a recombinant version of a biologically active cell-permeant human HAND2 transcription factor from *E. coli*

**DOI:** 10.1038/s41598-022-19745-w

**Published:** 2022-09-27

**Authors:** Krishna Kumar Haridhasapavalan, Pradeep Kumar Sundaravadivelu, Neha Joshi, Nayan Jyoti Das, Anshuman Mohapatra, Udayashree Voorkara, Vishwas Kaveeshwar, Rajkumar P. Thummer

**Affiliations:** 1 Laboratory for Stem Cell Engineering and Regenerative Medicine, Department of Biosciences and Bioengineering, Indian Institute of Technology Guwahati, Guwahati, 781039 Assam India; 2 Organelle Biology and Cellular Ageing Lab, Department of Biosciences and Bioengineering, Indian Institute of Technology Guwahati, Guwahati, 781039 Assam India; 3 Department of Biosciences and Bioengineering, Indian Institute of Technology Guwahati, Guwahati, 781039 Assam India; 4 Department of Obstetrics and Gynaecology, SDM College of Medical Sciences and Hospital, Shri Dharmasthala Manjunatheshwara University, Dharwad, 580009 Karnataka India; 5 Central Research Laboratory, SDM College of Medical Sciences and Hospital, Shri Dharmasthala Manjunatheshwara University, Dharwad, 580009 Karnataka India

**Keywords:** Biotechnology, Molecular engineering, Protein delivery

## Abstract

Transcription factor HAND2 has a significant role in vascularization, angiogenesis, and cardiac neural crest development. It is one of the key cardiac factors crucial for the enhanced derivation of functional and mature myocytes from non-myocyte cells. Here, we report the generation of the recombinant human HAND2 fusion protein from the heterologous system. First, we cloned the full-length human *HAND2* gene (only protein-coding sequence) after codon optimization along with the fusion tags (for cell penetration, nuclear translocation, and affinity purification) into the expression vector. We then transformed and expressed it in *Escherichia coli* strain, BL21(DE3). Next, the effect (in terms of expression) of tagging fusion tags with this recombinant protein at two different terminals was also investigated. Using affinity chromatography, we established the one-step homogeneous purification of recombinant human HAND2 fusion protein; and through circular dichroism spectroscopy, we established that this purified protein had retained its secondary structure. We then showed that this purified human protein could transduce the human cells and translocate to its nucleus. The generated recombinant HAND2 fusion protein showed angiogenic potential in the ex vivo chicken embryo model. Following transduction in MEF2C overexpressing cardiomyoblast cells, this purified recombinant protein synergistically activated the α-MHC promoter and induced GFP expression in the α-MHC-eGFP reporter assay. Prospectively, the purified bioactive recombinant HAND2 protein can potentially be a safe and effective molecular tool in the direct cardiac reprogramming process and other biological applications.

## Introduction

The role of recombinant proteins in therapeutics has been indispensable for the past four decades and will continue to be so. Production of recombinant proteins using different host organisms such as algae^1^, bacteria^2^, yeast^3^, insects^4^, and mammalian cells^5^ has so far proven to be a complex but effective process^6^. An immense number of recombinant proteins have been expressed, purified, and used for a wide range of biotechnological applications to date^7^. The most commonly used expression host system for recombinant protein production is the bacterial system, especially *Escherichia coli (E. coli)* strains due to easy handling and maintenance, well-studied genetics, well-understood cell machinery, high protein yield, and so forth^8–10^. Once expressed, these proteins are purified using a wide range of purification tags. The poly-histidine tag is the most widely used tag for purification since it is inexpensive and does not alter the characteristics of the proteins^11,12^. The introduction of recombinant proteins into mammalian cells has been proven to be an effective alternative since it does not integrate and alter the genome, and also manipulation of cell fate can be done in a time- and dosage-dependent manner^13–17^. Thus, recombinant proteins contribute to a major and vital part in therapeutics and in safer and non-integrative cell reprogramming processes. However, various challenges are associated with the successful production of these therapeutic recombinant proteins, such as codon usage bias, gene toxicity, mRNA instability, poor protein expression, proteolytic cleavage by the host cell, purity, poor solubility and stability (in vitro), and protein misfolding^13,17,18^. In this study, by circumventing these limitations, we aimed to generate a cell- and nuclear-permeant bioactive human Heart- and neural crest derivatives-expressed protein 2 (HAND2) protein that can prospectively be used for various biological applications.

HAND2 is a member of the basic helix-loop-helix family of transcription factors with a consensus DNA binding sequence 5’-CANNTG-3′^19^. The transcript of the *HAND2* gene is 2780 bp long, containing two exons. The coding sequence is 654 bp in length which translates to the protein of 218 amino acids. HAND2 is highly expressed in maternal decidua^20^ and adult heart, liver, and testes^20^. The knockout of the *HAND2* gene in mice leads to defects in ventricle formation, eventually leading to embryonic lethality^19,21^. A recent study reported HAND2 regulated genes involved in the atrioventricular canal and cardiac valve development^22^. HAND2 governs the development of epicardium, vascularization and angiogenesis, second heart field development and survival, and cardiac neural crest development^23^. All these studies indicate the role of HAND2 in cardiac development, both in mesoderm-derived and neural crest-derived structures.

Apart from these, HAND2 plays a critical role in the development of other tissues during mouse embryogenesis. It is known to have a significant role in the development of branchial arch and limb bud^21,24^. HAND2 regulates the anterior–posterior polarity of limb bud through a chain of downstream transcriptional regulators^24^. It also aids in craniofacial development^21^, and is essential for developing the sympathetic nervous system in humans, especially noradrenergic neurons^25^.

As HAND2 is crucial for the formation and maturation of cardiomyocytes^19^, it is regarded as one of the most crucial cardiac reprogramming factors for deriving functional cardiomyocytes. Several studies have used the genetic form of HAND2 in their reprogramming cocktail, and its inclusion resulted in higher efficiency than the original GATA4, MEF2C, and TBX5 combination^26–28^. The role of HAND2 in altering the chromatin accessibility and gene expression in fibroblasts to convert them to cardiac pacemaker-like cells has also been reported^29^.

Recently, we have demonstrated the heterologous expression and purification of human cardiac reprogramming factors, namely ETS2^30^, MESP1^31^, GATA4^32^, and TBX5^33^, in recombinant forms. Here, we have demonstrated the soluble expression and purification of recombinant human HAND2 (rhHAND2) protein from *E. coli* under native conditions having efficient cell permeability, nuclear translocation ability, and angiogenic potential. Using a GFP-based reporter assay, we demonstrated the transcriptional activity of this purified recombinant protein. To the best of our knowledge, this is the first study to identify the optimal induction parameters for the soluble expression and purification (native conditions) of this transcription factor, HAND2, in a bioactive form from *E. coli*.

## Materials and methods

### Construction of HAND2 fusion genetic constructs

The protein-coding sequence of the *HAND2* gene was retrieved, codon-optimized, evaluated, and cloned in a protein expression vector as depicted in Fig. S1. The resulting plasmids were verified by DNA sequencing and also by restriction digestion analysis using different combinations of restriction enzymes. The in silico online tools used for codon optimization and its evaluation are listed in Table S1. The primers used for sequencing are listed in Table S2.

### Identification of ideal expression parameters

*Escherichia coli* BL21(DE3) cells were used for all the expression analysis experiments and purification. The cells were transformed with appropriate recombinant plasmids harboring the *HAND2* fusion gene and cultured in Luria–Bertani broth (HiMedia) in the presence of antibiotic kanamycin (HiMedia), as described recently^32^. From that initial culture, the secondary culture was prepared with 2% inoculum in Terrific broth (HiMedia) in the presence of kanamycin (50 µg/mL). This culture was supplemented with 0.4% glycerol (Merck Millipore) and 1% glucose (HiMedia), and grown until it reached the desired cell density at 37 °C with continuous shaking at 180 rpm. The rhHAND2 fusion protein expression was then induced (in 20 mL of culture) with a required concentration of Isopropyl β-d-1-thiogalactopyranoside (IPTG) (HiMedia) and incubated for a respective time at a respective temperature depending on experimental requirements. The various values of the parameters such as IPTG concentration, pre-induction cell density, postinduction incubation time, and induction temperature screened for analysis are listed in Table S3. Uninduced cultures (20 mL) were incubated to confirm the absence of any leaky expression. After the incubation, the cells were harvested and lysed in prechilled lysis buffer (1.2 mL) (Table S4) by ultrasonication on ice. The obtained total cell lysate was clarified to separate the insoluble pellet and the soluble supernatant fractions. All the protein samples were analyzed using sodium dodecyl sulfate–polyacrylamide gel electrophoresis (SDS-PAGE) and immunoblotting.

### Immobilized metal ion affinity chromatography

From the soluble fraction, the rhHAND2 fusion protein was purified using Immobilized Metal Ion Affinity Chromatography under native conditions. Briefly, rhHAND2 fusion protein expression was induced in large culture volumes (600 mL) with optimized expression parameters. Cells were harvested, resuspended in lysis buffer (20 mL), and lysed using ultrasonication on ice. The total crude cell lysate was clarified by centrifugation to obtain a soluble supernatant fraction. The soluble fraction was incubated with nickel-nitrilotriacetic acid (Ni–NTA) resin at 4 °C for 8 to 14 h with continuous shaking. After incubation, the soluble fraction with Ni–NTA was loaded onto the purification column (Bio-Rad), equilibrated with lysis buffer, and the flow-through was drained out. Consequently, the column was washed with wash buffers (100 mL) sequentially. Once the wash buffers were fully drained out, elution buffer was applied to elute out the desired rhHAND2 protein. The PD10 columns (GE Healthcare) were used to desalt the purified protein according to the manufacturer’s instructions against glycerol buffer and stored at − 80 °C until further use. The purity of the rhHAND2 fusion protein was determined by SDS-PAGE, and identity was confirmed with immunoblotting. All the buffers used in this purification, along with their compositions, are listed in Table S4. The pH of the buffers was adjusted to 8.0 at room temperature and prechilled on ice before use.

### SDS-PAGE and immunoblotting

The protein concentration of sonicated and purified samples was estimated using the Bradford assay^34^. The SDS-PAGE and immunoblotting were performed as described in our recent studies^30,32^ and as detailed in the supplemental information (Extended “Materials and methods”). The antibodies used and their respective concentrations are listed in Table S5.

### Far-ultraviolet circular dichroism spectroscopy

Far-ultraviolet (UV) circular dichroism (CD) spectroscopy was carried out as described in our recent studies^30,32^ and as detailed in the supplemental information (Extended Materials and Methods). From the CD spectrum obtained, the secondary structure content of the purified HAND2 protein was estimated using the online tool, Beta Structure Selection (BeStSel; http://bestsel.elte.hu/index.php).

### MALDI-TOF

The purified protein was desalted in deionized Milli-Q water using a PD-10 column from GE healthcare and concentrated fivefold using a Thermo scientific three kDa MWCO protein concentrator. The protein was diluted two times in α-Cyano-4-hydroxycinnamic acid matrix solution. The molecular weight was accurately determined using the Bruker Autoflex speed MALDI-TOF mass spectrometer.

### Cell culture

HeLa and Human foreskin fibroblasts (HFF), BJ, (ATCC® CRL-2522™) were cultured as previously described^30,31^. HiFi™ human umbilical vein endothelial cells (HUVEC) were cultured on 0.5% gelatin-coated dishes in HiEndoXL™ endothelial cell expansion medium (HiMedia) under standard cell culture conditions. HEK293T cells were cultured in a growth medium containing 10% fetal bovine serum (FBS) and 1% penicillin–streptomycin solution (P/S) in high-glucose DMEM under standard cell culture conditions. H9C2 cardiomyoblast cells were cultured in a growth medium containing 10% FBS and 1% penicillin–streptomycin solution (P/S) in low-glucose DMEM under standard cell culture conditions. All the cell lines were passaged with trypsin–EDTA (Invitrogen) at ~ 80–90% confluency in the ratio of 1:4. HFF and HUVEC were procured from American Type Culture Collection, USA and HiMedia, India, respectively. All the other cell lines used were acquired from National Centre for Cell Science, India.

### HAND2 fusion protein transduction

Cells were adjusted to 5 × 10^4^ (for HFF) and 1 × 10^5^/well (for HeLa) with an appropriate growth medium and seeded in 24-well culture plates. Cells were grown 12–24 h at 37 °C with 5% CO_2_ under humidified culture conditions and then treated with protein transduction medium (DMEM, 2 or 5% FBS, 1% P/S, and optimal concentrations of purified rhHAND2 fusion protein or glycerol buffer as a control) and re-incubated for 4–6 h. Post-incubation, cells were washed with phosphate buffer saline and used for further analysis.

### Immunocytochemistry and microscopy

Immunocytochemistry and microscopy were performed as described recently^32^ and as detailed in the supplemental information (Extended “Materials and methods”). The antibodies and their respective concentrations used for immunocytochemistry are listed in Table S5. For microscopy, the images were acquired using an inverted fluorescence microscope (IX83, Olympus, Japan) equipped with a DP80 CCD camera. Samples were illuminated using a pE-300 white CoolLED light source. Cells were then counterstained with Hoechst 33342 (Invitrogen), and image stacks were acquired using the 20x/0.45NA objective at 2 μm intervals. Images were analyzed by CellSens dimension (Olympus) and Image J software.

### In vitro scratch migration assay

HUVECs were seeded at 1 × 10^5^ cells/well in a 12-well culture plate in endothelial cell expansion media and grown until 95% confluence was achieved. The confluent monolayers were scratched with a sterile pipette tip (0–20 μL), and the spent media was discarded. The scratched monolayers were rinsed with sterile PBS and then treated with rhHTN-HAND2 protein or an equivalent volume of vehicle control (in endothelial cell expansion media) or VEGF^35,36^ for 12 h. Images were captured at 20 × magnification using an inverted brightfield microscope (ZOE Fluorescent Cell Imager, Bio-Rad) at different time intervals. The images were exported in .tiff file format for the quantification of the scratched area using ImageJ (1.48v) software. The migration percentage was calculated using the following formula.

$$Migration\,\, \left(\%\right)=\frac{\left(initial\,\, area-final\,\, area\right)}{initial\,\, area}\times 100$$

### Chicken chorioallantoic membrane (CAM) assay

The CAM assay was performed as described previously^35,36^ with minor modifications. The 3–4 days old embryonated chicken eggs were directly obtained from the local chicken egg hatching unit. Chicken eggshells were gently cut open at the top like a window to expose the CAM layer. Paper discs soaked in purified protein or control solutions were placed directly on the blood vessel (one disc/egg CAM layer) and sealed with adhesive tape. Sealed eggs were then incubated for 12 h at 37 °C with 60% humidity. The exposed blood vessels were visualized under LCD Digital Stereomicroscope (2X) equipped with a 2MP camera, and images were captured at specific intervals, followed by documentation with the help of Adobe Photoshop CC 2019 software.

### Lentivirus production

Second-generation lentiviral vectors were packaged in HEK293T cells using the calcium phosphate transfection method. Briefly, 6 × 10^6^ HEK293T cells were seeded in a 100 mm gelatin-coated dish and then grown to 65–75% confluence in the complete growth medium. Once the culture attained the desired confluence, the medium was replaced with a transfection medium containing advanced DMEM (Invitrogen) supplemented with 2% FBS, 1 × P/S, and 1 × non-essential amino acids (NEAA; Invitrogen), 1–2 h prior to transfection. The calcium phosphate transfection mixture was then prepared by mixing 12 μg lentiviral backbone plasmid [α-MHC-eGFP (Addgene plasmid 21229) or FUdeltaGW-rtTA (Addgene plasmid 19780) or tetO-MEF2C (Addgene plasmid 46031)], 7.7 μg psPAX2 (Addgene plasmid 12260), 4.3 μg pMD2.G (Addgene plasmid 12259), 125 mM CaCl_2_, and makeup with tissue-culture grade water and mixed with 2X HBS buffer (1:1), and incubated for 15 min at room temperature. This mixture was then added to the cells dropwise in the presence of 25 μM chloroquine. After 5–6 h of transfection, the medium was replaced with a virus production medium containing advanced DMEM supplemented with 5% FBS, 1 × P/S, and 1 × NEAA. The medium was then replaced with a fresh medium after 12–16 h post-transfection. The lentiviral supernatant was then harvested at 24, 36, and 48 h of incubation, pooled, concentrated (centrifugation at 14,000 rpm for 2 h), and aliquoted for storage at − 80 °C.

### α-MHC reporter assay

An outline of the experimental protocol is shown in Fig. 9A. Briefly, H9C2 cells were seeded in a gelatin-coated 12-well culture plate at 1 × 10^5^ cells/well and grown until 60–70% confluence was achieved. Cells were then transduced with α-MHC-eGFP [~ 5 × 10^5^ Infectious Units (IFU)] and FUdeltaGW-rtTA (~ 5 × 10^5^ IFU) lentiviral vectors^28^. On the next day (D-1), cells were infected with tetO-MEF2C (~ 5 × 10^5^ IFU). Cells were transduced only in the presence of 5 µg/mL of polybrene. On the following day (D0), cells were reseeded in the ratio of 1:3 in a fresh gelatin-coated 12-well culture plates. On Day 1, the spent medium was replaced with a 5% FBS growth medium supplemented with 2 µg/mL of doxycycline (Sigma). The next day (D2), cells were washed with phosphate buffer saline and then treated with a protein transduction medium containing 400 nM of rhHAND2 fusion protein or an equivalent volume of vehicle control supplemented with 2 µg/mL of doxycycline. The medium was renewed every alternative day. After the treatment, images were captured in the green channel at 20 × magnification using an inverted fluorescent microscope (ZOE Fluorescent Cell Imager, Bio-Rad).

### Flow cytometry

Cells were harvested from culture dishes and washed with PBS for GFP expression analyses, followed by fixation with 4% paraformaldehyde for 15 min. Fixed cells were washed and resuspended in phosphate buffer saline and then analyzed using a BD FACS Calibur Flow Cytometer (BD Biosciences) with FlowJo software.

### Statistical analysis

The experimental data obtained were analyzed by *t-*test using GraphPad Prism 8 software. Values are expressed as mean ± SEM, and P < 0.05 was considered significant.

## Results

### Codon optimization and cloning of human HAND2 gene sequence

We first codon-optimized the non-optimized *HAND2* protein-coding sequence for expression in *E. coli*. The alignment of non- and codon-optimized codons and their respective amino acids is depicted in Fig. S2. Before and after codon optimization, the nucleotide sequence was analyzed using two different online tools for comparison. The Genscript Rare Codon Analysis (GRCA) and Graphical Codon Usage Analyzer (GCUA) tools showed that 7% (Fig. S3; Table S6) and ~ 3% (Fig. S4) of the codons, respectively, were rare codons before optimization. After optimization, analysis using these tools revealed that these rare codons were replaced with the most abundant codons in *E. coli*. Moreover, codon adaptation index value improved from 0.69 for non-optimized to 0.89 for codon-optimized sequence (Table S6). Ideally, the codon adaptation index value should lie between 0.8 and 1.0 (Table 1), which indicates that the optimized sequence of *HAND2* is ideal for expression in *E. coli*.

**Table 1:** Purification summary of recombinant human HTN-HAND2 protein.

Induction temperature (°C)	Steps	Total protein (mg)^b^	Target protein (mg)^c^	Yield (%)	Purity (%)^d^
37	Crude lysate ^a^	58.89	10.40	100	17.67
	Cleared lysate (soluble)	55.30	9.88	95	17.87
	IMAC (pooled)	0.88	0.87	4.13	99.01
18	Crude lysate ^a^	54.65	9.13	100	16.70
	Cleared lysate (soluble)	50.76	7.65	83.79	15.07
	IMAC (pooled)	0.62	0.60	5.37	97.92

^a^From 1 g wet weight of induced *E. coli* BL21(DE3) cell pellet (from 0.25 to 0.35 L of culture).

^b^Protein concentration determined by Bradford assay using BSA as a standard protein.

^c^Determined from total protein concentration^b^ and purity^d^.

^d^Purity determined from SDS-PAGE analysis using ImageJ software.

**Figure 1:**
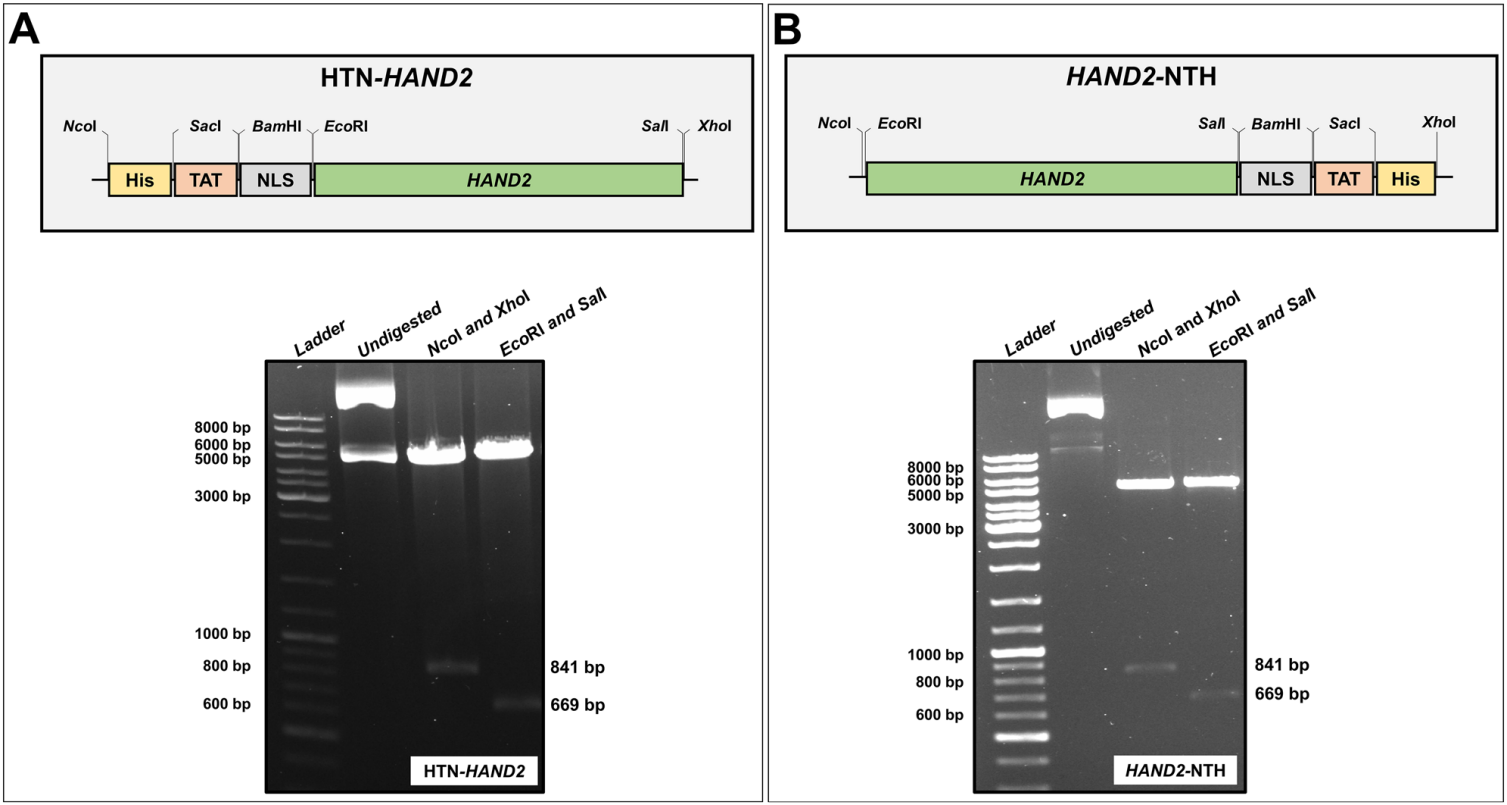
Schematic illustration of the tagging pattern in the human *HAND2* gene and verification of cloning. **(A)** An Illustration of HTN-*HAND2* (*top*) gene insert (not drawn to scale) and verification of the cloned plasmid pET28a(+)-HTN-*HAND2* (*bottom*) using restriction digestion analysis. (**B)** Illustration of *HAND2*-NTH (*top*) gene insert (not drawn to scale) and verification of the cloned plasmid pET28(+)-*HAND2*-NTH (*bottom*) using restriction digestion analysis. His (H): polyhistidine (8X); TAT (T): Transactivator of Transcription; NLS (N): Nuclear Localization Sequence/Signal. The uncropped gels are presented in Supplementary Figs. S6 and S7.

Next, we tagged this codon-optimized *HAND2* gene sequence with a set of fusion tags (His, TAT, and NLS) at either 5′ or 3′ end to generate *HAND2* fusion gene inserts [Fig. 1A,B (*top*)]. Various studies have reported that the expression, solubility, and stability of human proteins heterologously expressed in *E. coli* are influenced by the position of the fusion tags^30–33,37–43^. These fusion gene inserts (HTN-*HAND2* and *HAND2*-NTH) were artificially synthesized and subcloned into the pET28a(+) expression vector containing a tightly regulated T7 promoter (inducible). Restriction digestion with selected restriction enzymes and subsequent fragments on agarose gel verified the successful cloning of the *HAND2* fusion gene inserts into the vector [Fig. 1A,B (*bottom*); Table S7]. Further, we confirmed the successful cloning of the *HAND2* fusion genes in the proper orientation and the fidelity of its nucleotide sequences by DNA sequencing (data not shown). Thus, we developed the recombinant pET28a(+) plasmids harboring *HAND2* fusion genes [pET28a( +)-HTN-*HAND2* and pET28a(+)-*HAND2*-NTH constructs], which can be used to produce rhHAND2 fusion proteins (rhHTN-HAND2 and rhHAND2-NTH) with subcellular and subnuclear localization ability.

### Screening various parameters for obtaining soluble expression of the rhHAND2 fusion protein

**Figure 2:**
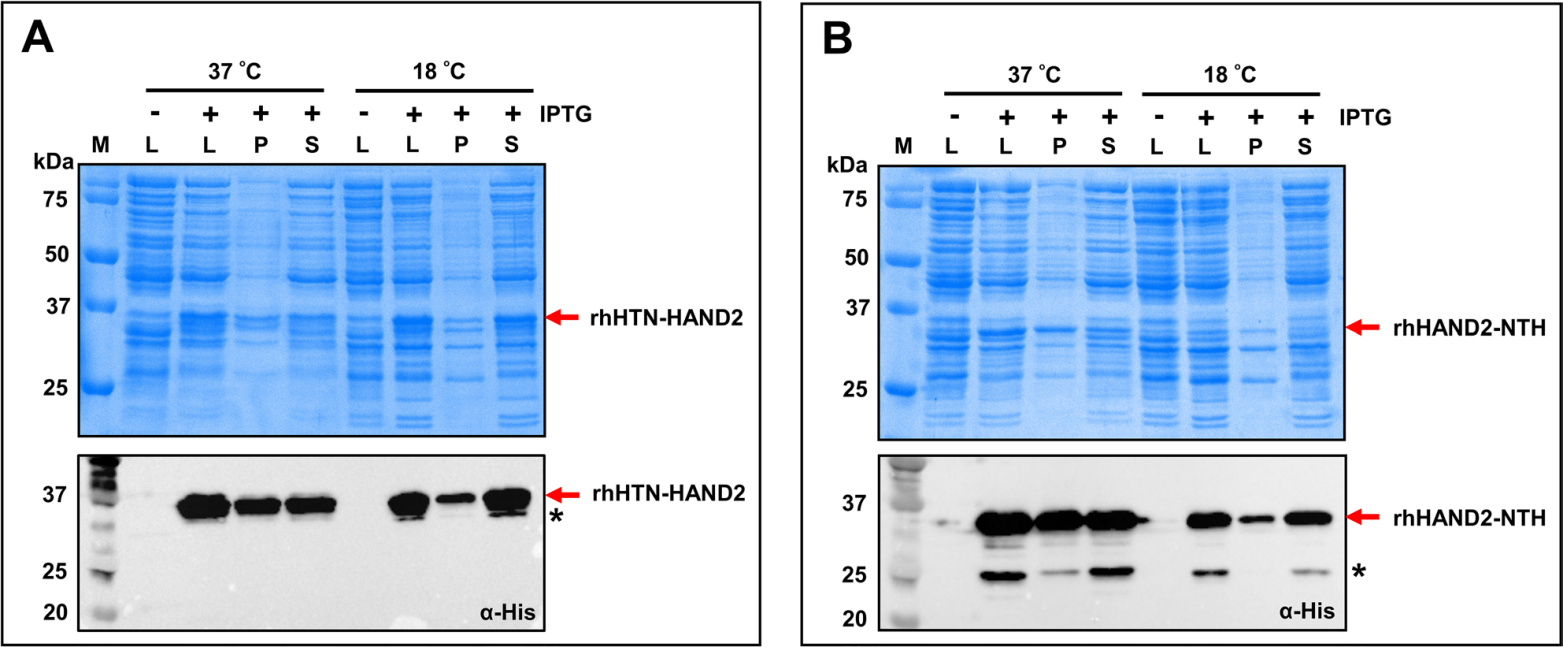
Analyzing the effect of induction temperature and tagging pattern on the expression of rhHAND2 fusion protein. *E. coli* BL21(DE3) cells were transformed with pET28a(+)-HTN-*HAND2* (**A)** and pET28a(+)-*HAND2*-NTH (**B)** and then induced the recombinant protein expression either at 37 °C for 2 h or 18 °C for 24 h. The cell lysate, soluble, and insoluble cell fractions obtained by lysing the cells were analyzed by SDS-PAGE (*top*) and immunoblotting (*bottom*) with normalized loading. (n = 2). *Truncations of the HAND2 fusion protein. *M* protein marker (kDa); *L* total cell lysate; *P* pellet/insoluble cell fraction; *S* supernatant/soluble cell fraction; *Ab* antibody. The uncropped gels and the blots are presented in Supplementary Figs. S8 and S9.

We next sought to determine optimal parameters for obtaining maximal soluble expression. Numerous studies have identified the optimal (ideal) conditions for achieving the maximal soluble expression of bioactive recombinant proteins^18,44–50^. In the process, we screened various parameters, namely pre-induction cell density, inducer concentration, and postinduction incubation time. The different values that were screened for each parameter are listed in Table S3. Firstly, from the screening experiments, optimal pre-induction cell density, inducer concentration, and postinduction incubation time was found to be ~ 0.5 OD_600_, 0.05 mM, and 2 h, respectively. We further examined the soluble expression of both the fusion proteins (rhHTN-HAND2 and rhHAND2-NTH) induced at 37 °C, using SDS-PAGE and immunoblotting. These results (Fig. 2A,B) revealed that in both the cases (rhHTN-HAND2 and rhHAND2-NTH), nearly half of the overall expressed HAND2 protein molecules are found in pellet/insoluble cell fractions and rest in the supernatant/soluble cell fractions. Earlier reports recommended that reduction in induction temperature enhances the protein solubility^31,44,46,49^. Therefore, we investigated whether the reduction in temperature enhances the solubility of the rhHAND2 fusion protein. This is crucial to avoid purification from inclusion bodies, which contain partially folded or unfolded protein of interest and require strong detergents to solubilize the inclusion bodies and extract the protein of interest and then refold to its native state^18,51^. In both cases, induction at 18 °C considerably improved the solubility of the rhHAND2 fusion protein (Fig. 2A,B). A decline in the overall expression only in rhHAND2-NTH fusion protein was observed when induced at 18 °C compared to 37 °C. The immunoblotting analysis further represents the truncated protein fragments of rhHAND2-NTH protein in both the induction temperatures, unlike rhHTN-HAND2 [Fig. 2A,B (*bottom*)]. These truncated fragments could be due to various possible reasons, such as (i) intragenic sequences that mimic *E. coli* ribosomal entry sites present within the protein-coding sequence^52^, (ii) proteolysis of some protein molecules at specific sensitive sites during expression^53^, (iii) protein cleavage at Asp-Pro bonds because of overheating of protein samples^54^. Among the three reasons, the last reason is least likely in this study as the protein samples of both rhHTN-HAND2 and rhHAND2-NTH were treated similarly, and no such truncations were observed in the case of rhHTN-HAND2. Importantly, these truncations might compromise the full-length rhHAND2 fusion protein quality and purity at the final stages. This observation signified the importance of induction temperature and the terminal at which the fusion tags were coupled in the production of quality recombinant proteins. An earlier study also reported similar observations with different transcription factors^37^. The overall identified optimal expression parameters are listed in Table S3. Thus, based on this analysis, fusion tags at the N-terminal end of HAND2 (rhHTN-HAND2) induced at 37 or 18 °C, were selected for further experiments.

### One-step purification of the rhHAND2 fusion protein

**Figure 3:**
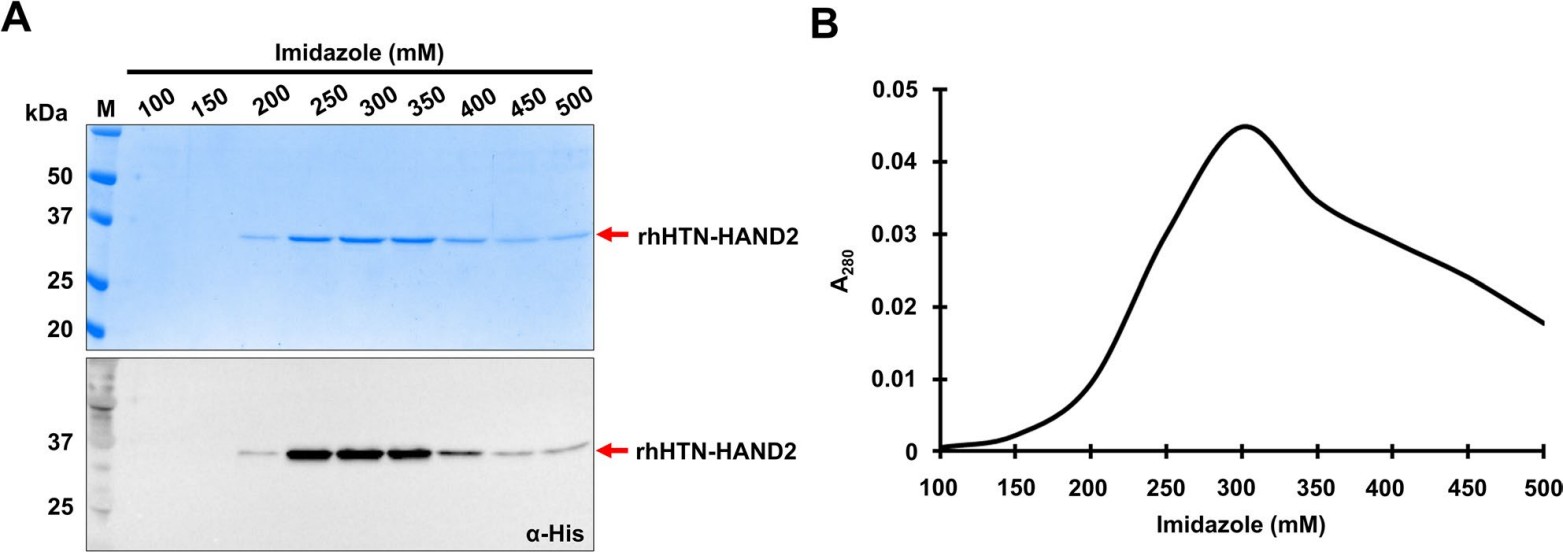
Effect of imidazole concentration on the amount of HAND2 protein eluted. (**A**) The expressed rhHTN-HAND2 protein was purified using affinity chromatography**.** During purification, the imidazole concentration in the elution buffer was varied to see its effect on the amount of protein eluted. As mentioned in the figure, different concentrations of imidazole were used in the elution buffer, and the protein was eluted. The eluted proteins were analyzed using SDS-PAGE (*top*) and immunoblotting (*bottom*) using an α-his antibody. (**B**) The absorbance of the protein eluted using different concentrations of imidazole in elution buffer was measured at 280 nm, and a graph was plotted with imidazole concentration on the x-axis against absorbance at 280 nm on the y-axis. The uncropped gel and the blot are presented in Supplementary Fig. S10.

To purify rhHTN-HAND2 protein from soluble cell fraction, IMAC (in this study, Ni^2+^-NTA resin) under native conditions was performed to retain the native-like secondary structure conformation of this fusion protein. Markedly, proteins purified under native (soluble cell fraction) conditions often produced bioactive molecules with native-like folding conformations^55^. At first, the effect of imidazole concentration on the elution of rhHAND2 fusion protein-induced at 37 °C was investigated. As shown in Fig. 3A, this recombinant fusion protein started eluting with 200 mM of imidazole, and the maximum amount was eluted with 250–350 mM. Importantly, no bacterial proteins were observed in any of the elution. The imidazole gradient elution profile of affinity-purified rhHAND2 protein, induced at 37 °C, is shown in Fig. 3B. Thus, for the one-step purification of the rhHAND2 fusion protein, induced at two different temperatures, we used a maximum of 150 mM of imidazole during washing and 300 mM for eluting the rhHAND2 fusion protein.

**Figure 4:**
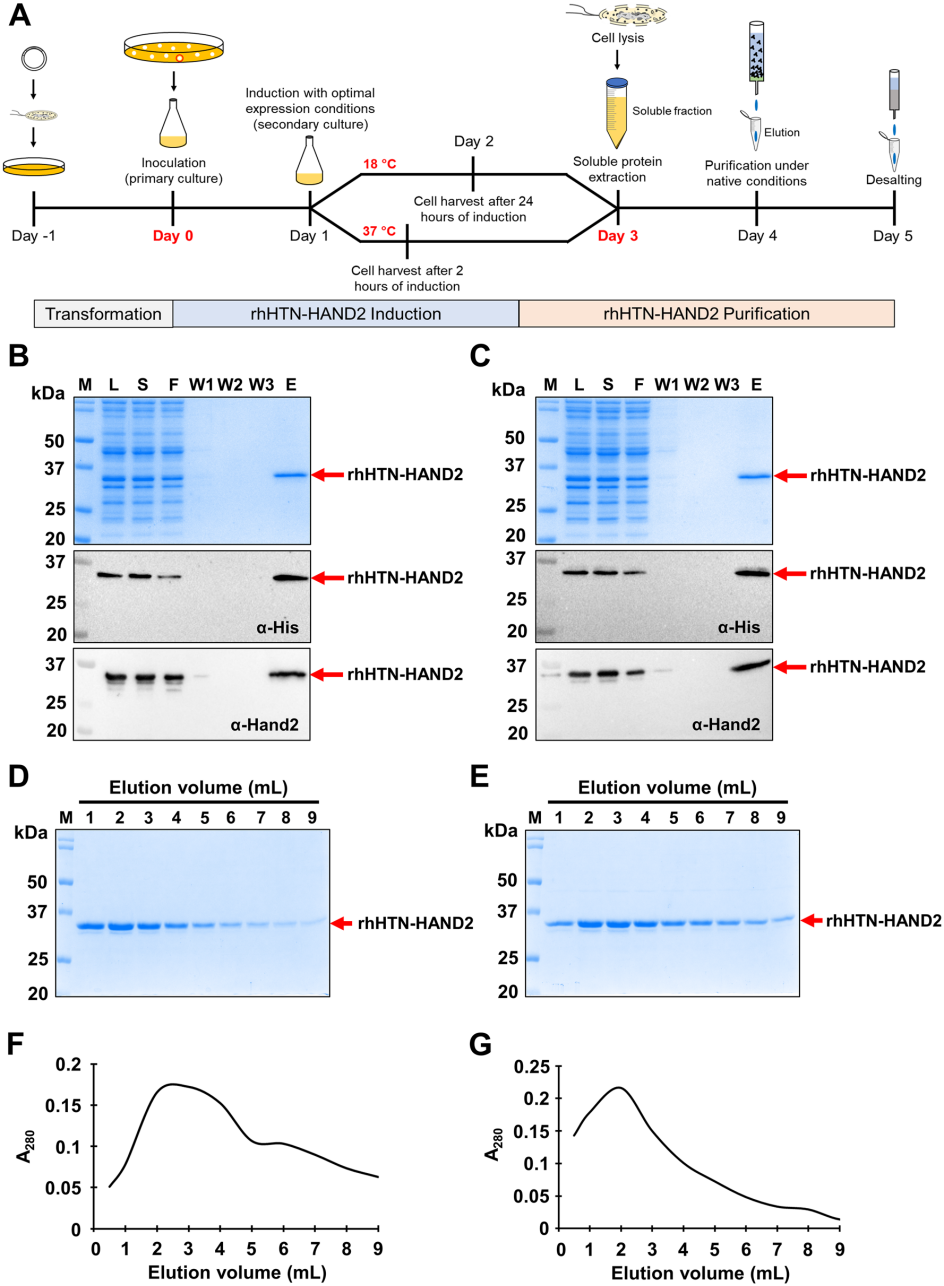
One-step purification of rhHAND2 protein, induced at two different temperatures. (**A**) Timeline and pictorial representation of the overall experimental strategy. (**B**,**C**) The purification of rhHTN-HAND2 protein at two different induction temperatures, 37 (n = 11) and 18 °C (n = 9), respectively. The samples collected during different stages of purification were analyzed using SDS-PAGE (*top*) and immunoblotting using α-his (*middle*) and α-Hand2 (*bottom*) antibodies. 20 µg/lane for L fraction, equal volumes for S-W3 fractions corresponding to L fraction, and 40 µL of E fraction were used for the analysis (n ≥ 9). (**D**,**E**) Analysis of elution fractions of the purified rhHAND2 fusion protein-induced at 37 and 18 °C, respectively. The expressed rhHTN-HAND2 protein, induced at two different temperatures, was purified, and the eluted proteins were analyzed using SDS-PAGE gel with equal loading volume. (**F**,**G**) Elution profile of one-step homogeneous purification of the rhHAND2 fusion protein-induced at 37 and 18 °C, respectively. *M* protein marker, *L* lysate, *S* soluble supernatant, *F* flow-through, *W1* wash buffer 1, *W2* wash buffer 2, *W3* wash buffer 3, *E* elution. The uncropped gels and the blots are presented in Supplementary Figs. S11, S12, S13, and S14.

With the identified expression conditions, we expressed and purified the rhHTN-HAND2 protein under native conditions, as shown in Fig. 4A. From the SDS-PAGE and immunoblotting analysis, it is clear that the rhHTN-HAND2 protein, induced at two different temperatures, 37 and 18 °C, has been purified without bacterial contaminants (Fig. 4B,C). A single band (~ 34 kDa) in the eluted fractions was observed on SDS-PAGE, indicating the high purity of rhHAND2 fusion protein [Fig. 4B (*top*), 4C (*top*), 4D and 4E]. This is crucial because purified proteins having bacterial proteins are likely to induce undesired effects on the target human cells when applied to elucidate their biological function^56^. Immunoblotting analysis revealed the loss of rhHAND2 fusion protein (Fig. 4B,C) in the flow-through/unbound fraction. This could probably be due to the overloading of the soluble fraction on the purification column or the low volume of resin used for purification. The identity of this fusion protein was established by immunoblotting with the anti-Hand2 antibody [Fig. 4B,C (*bottom*)]. Interestingly, the purified rhHTN-HAND2 protein was eluted efficiently only from the second elution fraction (Fig. 4D,E), signifying that the interactions between the protein molecules and Ni–NTA were stronger and required more elution with elution buffer to weaken these interactions before eluting. The elution profile of one-step affinity-purified rhHAND2 fusion proteins induced at 37 °C and 18 °C is shown in Fig. 4F,G respectively. The purification data are summarized in Table 1, and the final yield of the purified rhHAND2 fusion protein was around 0.87 and 0.60 mg/g of wet cells when induced at 37 and 18 °C, respectively. Thus, the one-step purification of rhHAND2 fusion protein under native conditions from soluble fractions was demonstrated, irrespective of the induction temperatures.

### MALDI-TOF and secondary structure analysis of rhHTN-HAND2 protein

**Figure 5:**
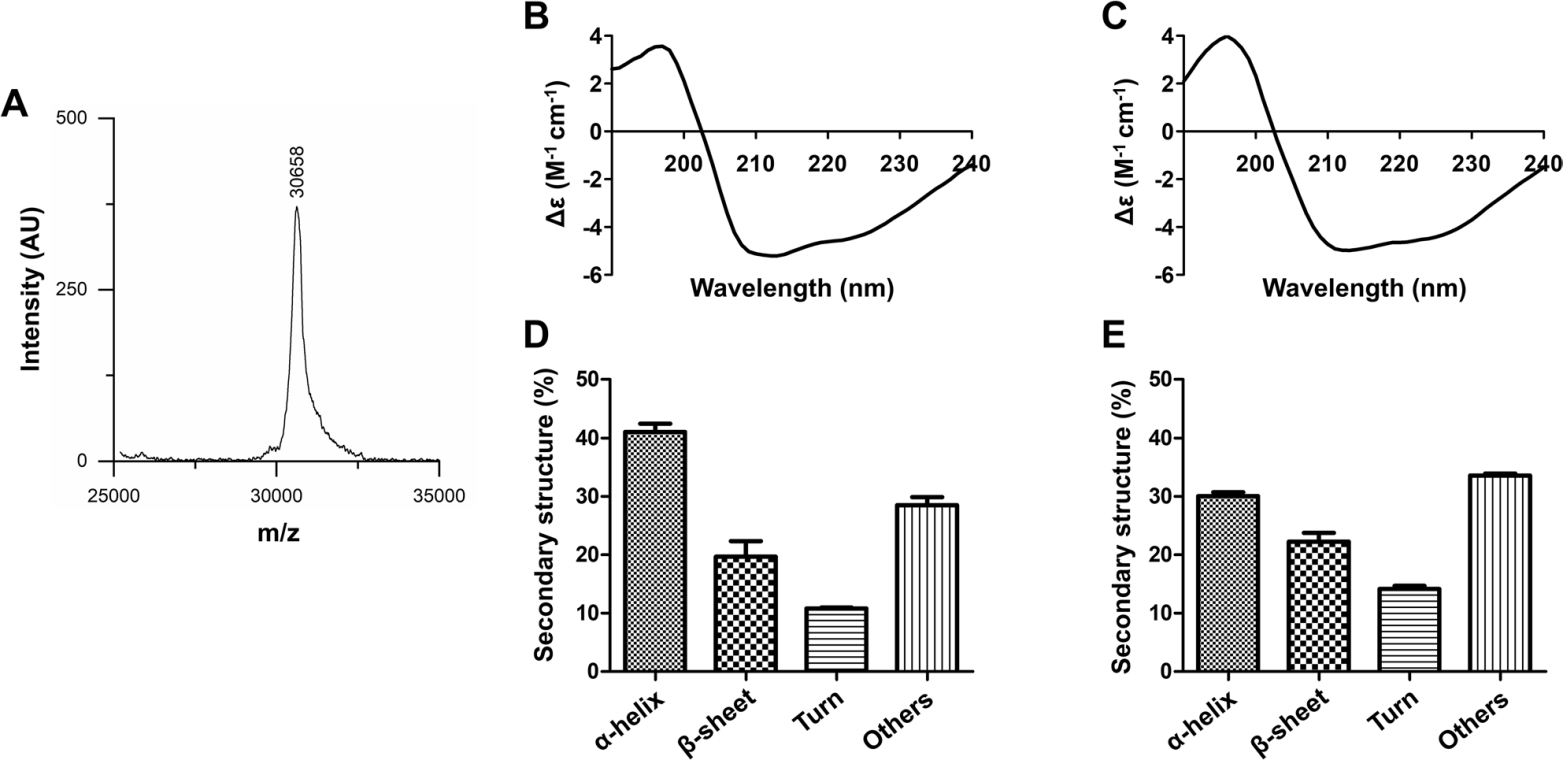
MALDI-TOF and Circular dichroism spectroscopy analysis of the purified rhHAND2 fusion protein. (**A**) The MALDI-TOF–MS analysis of the purified HAND2 protein. The purified rhHAND2 fusion protein was analyzed using MALDI-TOF, and the result is depicted using a graph with mass per charge (m/z) ratio on the X-axis and intensity (AU) on the Y-axis. (**B**–**E**) The secondary structure content of this purified rhHAND2 fusion protein was determined using far-UV CD spectroscopy. The obtained far-UV CD spectra were then evaluated using in silico BeStSel online tool. (**B**,**C**) The CD spectrum of the purified rhHAND2 fusion proteins induced at 37 and 18 °C, respectively, were plotted with Delta Epsilon (M^−1^ cm^−1^; Y-axis) against wavelength (nM; X-axis). (**D**,**E**) The bar graphs represent the quantified secondary structure of the purified rhHTN-HAND2 proteins induced at 37 and 18 °C, respectively.

The monoisotopic molecular weight of the rhHAND2 fusion protein 30,687.805 Da was calculated from the amino acid sequence using the Peptide Mass Calculator online server (https://www.peptidesynthetics.co.uk/tools/). The molecular weight was accurately determined using MALDI-TOF mass spectrometer and found to be 30,658 Da (Fig. 5A). The difference in the observed and calculated molecular weight remains well within the calibration error of the instrument. We next sought to study the secondary structure content of the purified rhHAND2 protein, as the crystal structure of this human protein has not been reported yet. Commonly, the CD spectroscopic technique is used to study the folding conformation/characteristics of desired proteins whose secondary structure is unknown^57,58^, like human HAND2. Principally, different secondary structures absorb different amounts of right and left circularly polarized light. Thus, the spectrum obtained from the CD technique is characteristic in shape and magnitude to the specific type of secondary structure of the protein, namely α-helices, β-sheets, turns, and random coils^57,58^. Typically, in a CD spectrum, α-helices show two negative peaks at 222 and 208 nm and a single positive peak at 193 nm^57^. Correspondingly, β-sheets have a negative peak at 218 nm and a positive peak at 195 nm, whereas random coils have a negative peak at 195 nm and a positive peak at 210 nm^57^. The overall spectra are obtained depending on the various amounts of secondary structure content present in the given protein.

From the CD spectra of purified rhHAND2 protein, induced at two different temperatures, 37 °C and 18 °C (Fig. 5B,C), it is evident that this protein has maintained its secondary structure. Moreover, the level of similarity between both spectra indicates that the secondary structure is consistent irrespective of the induction temperature. From the CD spectra, different secondary structures were quantified using BeStSel online tool. The secondary structure content of rhHAND2 protein, induced at two different temperatures, 37 °C and 18 °C (Fig. 5D,E), revealed that this purified protein comprises majorly of α-helices and other structures (mostly random coils). From the results (Fig. 5D,E), it is apparent that 41% of α-helices and 28% of other structures were observed when the induction temperature was 37 °C, whereas 30% of α-helices and 34% of other structures were observed when the induction temperature was 18 °C. Furthermore, there is a significant contribution of β-sheets (20% (induced at 37 °C) and 22% (induced at 18 °C)) and turns (11% (induced at 37 °C) and 14% (induced at 18 °C)) to the secondary structure of HAND2 protein. Although the spectra appeared similar, variability in the secondary structure content was observed upon the quantification using BeStSel analysis. This variability is likely due to the induction of rhHTN-HAND2 at different temperatures, which might have resulted in differences in their folding. The possible minor differences in the protein concentrations (37 °C v/s 18 °C) may also be the reason for this variability. Due to the non-availability of the 3D structure of human HAND2 protein, the spectra and the secondary structure content determined in this study may be slightly different from the native protein, or the secondary structure content of the native protein may be similar to either 37 °C or 18 °C. Further comparison can be made once the crystal structure of full-length human HAND2 is available. The presence of fusion tags may also alter the structure of the HAND2 protein, and thereby the secondary structure content estimated in this study may be slightly different from the native protein. In general, our results have established that this purified rhHTN-HAND2 protein has maintained its secondary structure post-purification.

### Effect and transduction ability of recombinant form of the purified HAND2 fusion protein in human cells

**Figure 6:**
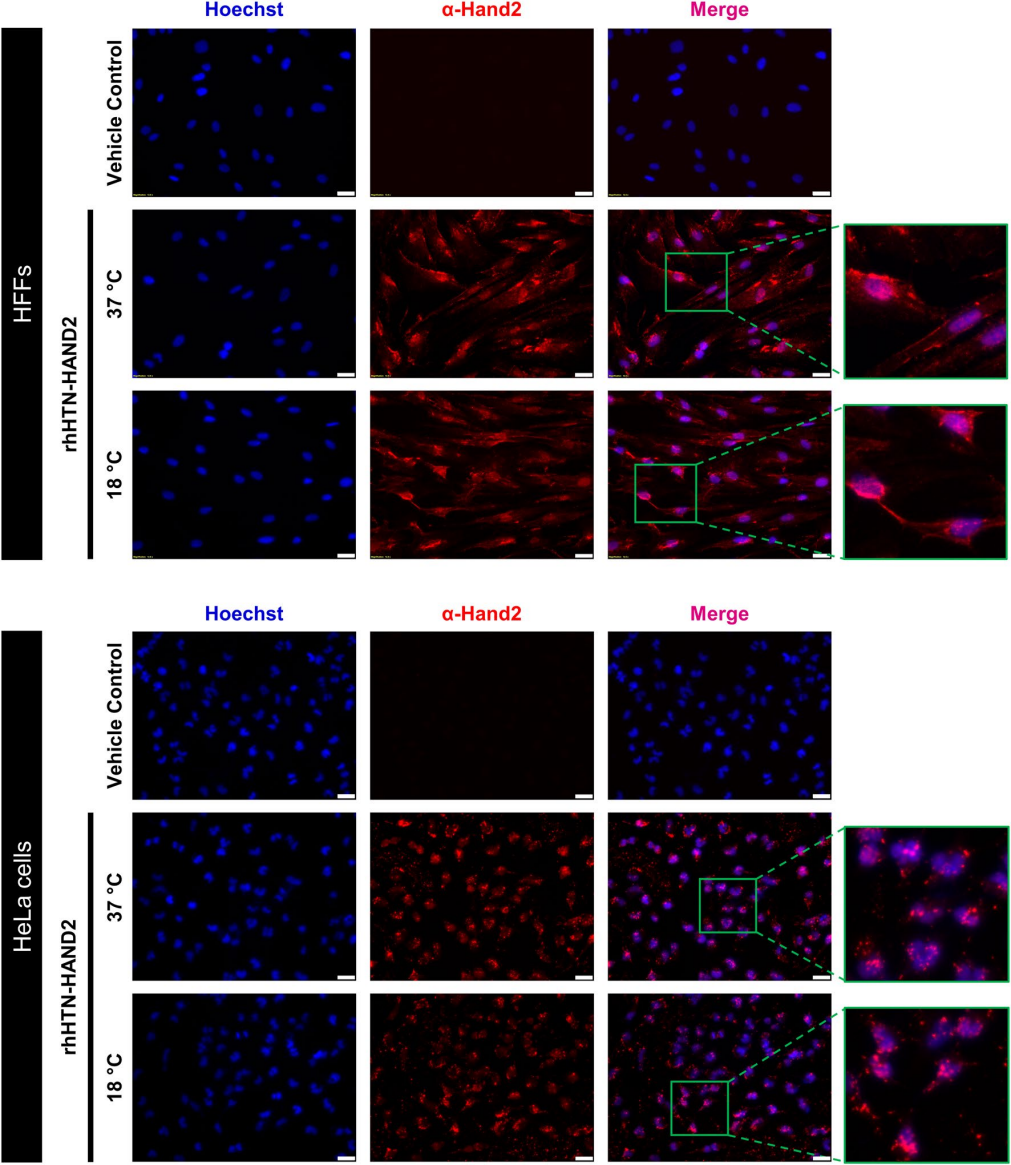
Subcellular and subnuclear delivery of purified rhHTN-HAND2 protein into human cells. HFF and HeLa cells were exposed to 200 nM of purified rhHTN-HAND2 protein for 4–6 h under standard cell culture conditions. After protein transduction, cells were fixed, permeabilized, blocked, and then stained with an α-Hand2 antibody. Transduced cells were detected with an Alexa Fluor® 594 conjugated α-rabbit secondary antibody. Nuclei were stained with Hoechst, and images were taken at ×20 magnification. All images were taken with identical camera settings. Scale bar: 50 μm (n = 3).

We next investigated the transduction ability of this purified fusion protein in human cells. BJ fibroblasts and HeLa cells were exposed either with 200 nM of rhHTN-HAND2 protein, induced at two different temperatures, 37 and 18 °C, or glycerol buffer as a negative control (vehicle control) for 4–6 h and then analyzed using fluorescence microscopy. BJ and HeLa cell lines were chosen for protein transduction analysis because they lack endogenous expression of HAND2 protein. Also, BJ human fibroblasts were selected since they are widely used for cellular reprogramming studies^13,14^, and HeLa cells are commonly used for transduction studies^33,43^. As shown in Fig. 6, the transduction of rhHAND2 fusion protein across the sub-cellular and sub-nuclear regions of both cell lines was observed. The microscopy results further confirmed the absence of endogenous expression of HAND2 in BJ fibroblasts and HeLa cells as shown in the vehicle control panel, and this also signifies that the glycerol buffer does not trigger the expression of HAND2 or did not lead to any false positive signal during analysis (Fig. 6). Thus, this purified recombinant fusion protein (irrespective of the induction temperature in which they were expressed) has cell penetration (mediated by TAT) and nuclear translocation (mediated by NLS) ability, indicating the functionality of these tags post-purification.

### Effect of purified rhHTN-HAND2 protein on endothelial cell migration

**Figure 7:**
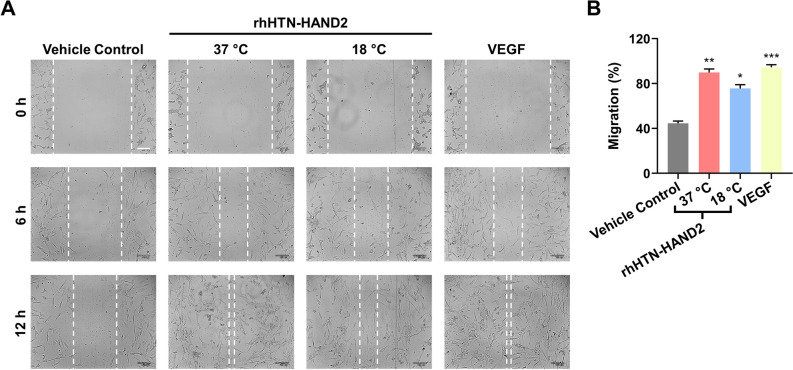
Effect of purified rhHAND2 fusion proteins on the migration of endothelial cells. (**A**) Microscopy analysis of the migration-inducing potential of rhHTN-HAND2 proteins transduced, non-transduced, and VEGF induced HUVECs. Scale bar: 100 μm. (**B**) Quantitative data of the migration in (**A**). The quantitative data shown are mean ± SEM (n = 3). *P < 0.01; **P < 0.001, ***P < 0.0001.

Although this purified protein has retained its secondary structure, further examination is required to corroborate its functional activity. Therefore, to validate the biological activity of this purified fusion protein, we first investigated its effect on the migration potential of endothelial cells. Previously, it has been reported that HAND2 plays a crucial role in regulating angiogenesis^23,59^. Endothelial cell migration is one of the important processes of angiogenesis. In that aspect, we observed that this purified rhHAND2 fusion protein promotes endothelial cell migration. After 12 h of treatment, protein transduced HUVECs migrated to > 75% scratched area, whereas in non-transduced vehicle control cells, only less than half the area was covered (Fig. 7A,B). The rhHTN-HAND2 protein-induced at 37 °C migrated faster than that induced at 18 °C. Interestingly, rhHTN-HAND2 protein-induced at 37 °C promotes endothelial cell migration similar (no significant difference) to that of VEGF treated ones (Fig. 7B). These results thus show that the purified proteins are functional.

### Angiogenic potential of purified rhHTN-HAND2 protein using ex vivo chick embryo model

**Figure 8:**
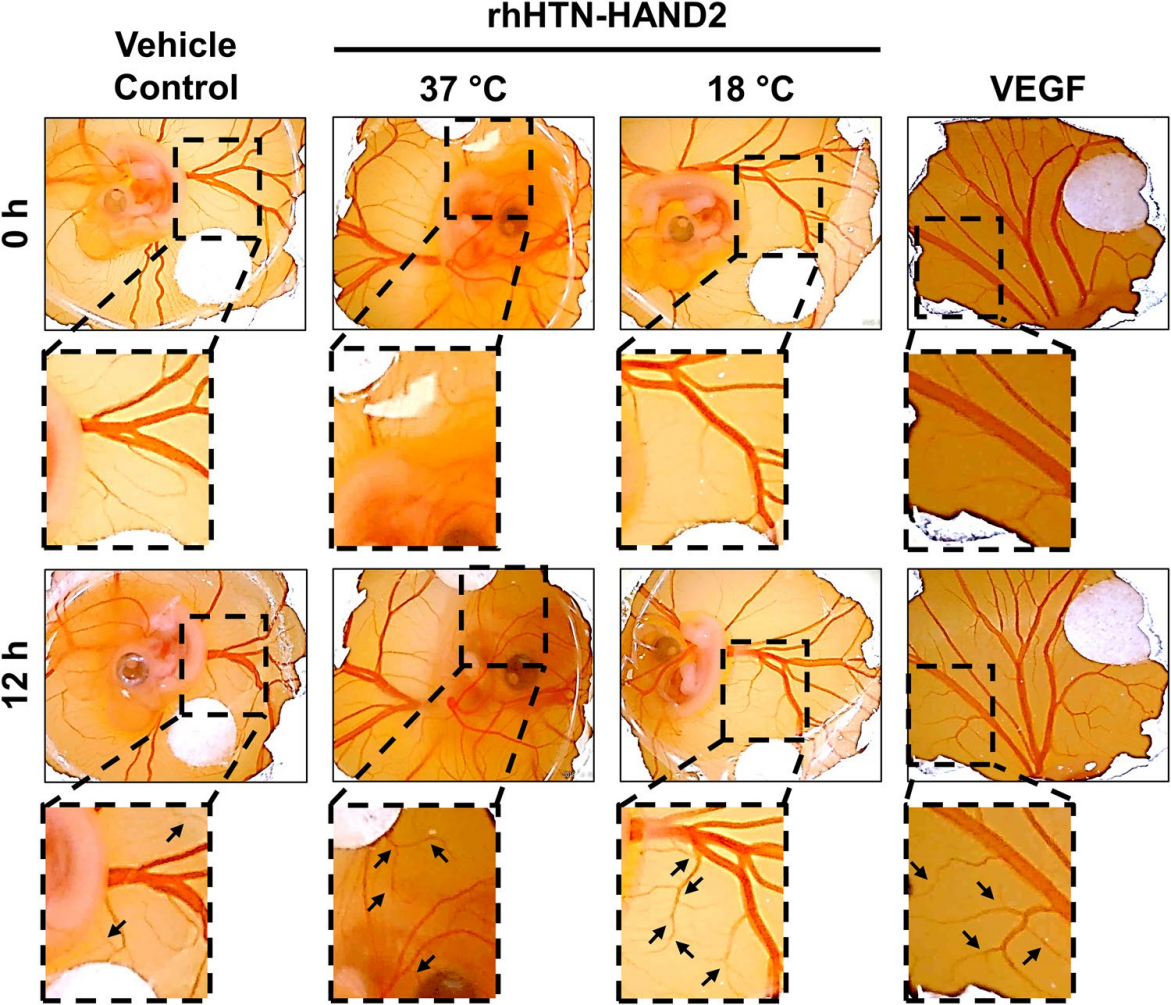
Examination of angiogenic potential of the purified rhHAND2 fusion protein using ex vivo chicken CAM model. Embryonated chicken eggs 3–4 days old were gently cut open on the top and treated with purified rhHTN-HAND2 protein (induced either at 37 or 18 °C) or vehicle control or VEGF (positive control) for 12 h at 37 °C. Macroscopic images of CAM were captured before and after the incubation. All images were taken with identical camera settings. Images are representative of three different experiments.

We next sought to understand whether this purified HAND2 protein can promote angiogenesis as we obtained positive results in the preliminary experiments using endothelial cells. In order to study the angiogenic potential of HAND2 protein, we performed a chicken CAM assay. The chick embryo model is a widely used ex vivo model to study the angiogenic/anti-angiogenic potential of various biological molecules including recombinant proteins^35,36^. For the slow diffusion of recombinant protein, we soaked the filter paper discs in the protein solution and gently placed them on the CAMs. At the end of 12 h of incubation with rhHTN-HAND2 protein, we observed the induced sprouting of small capillaries from the pre-existing blood vessels (Fig. 8; shown by arrows). Interestingly, independent of the induction temperature, increased neovascularization was observed in both the fusion proteins and VEGF (positive control) compared to the vehicle control. However, no significant difference was observed between both the fusion proteins and VEGF (Fig. 8). Thus, these results confirm that the purified rhHTN-HAND2 protein induces neovascularization by sprouting angiogenesis.

### Activation of the α-MHC promoter by rhHTN-HAND2 protein in the presence of MEF2C

**Figure 9:**
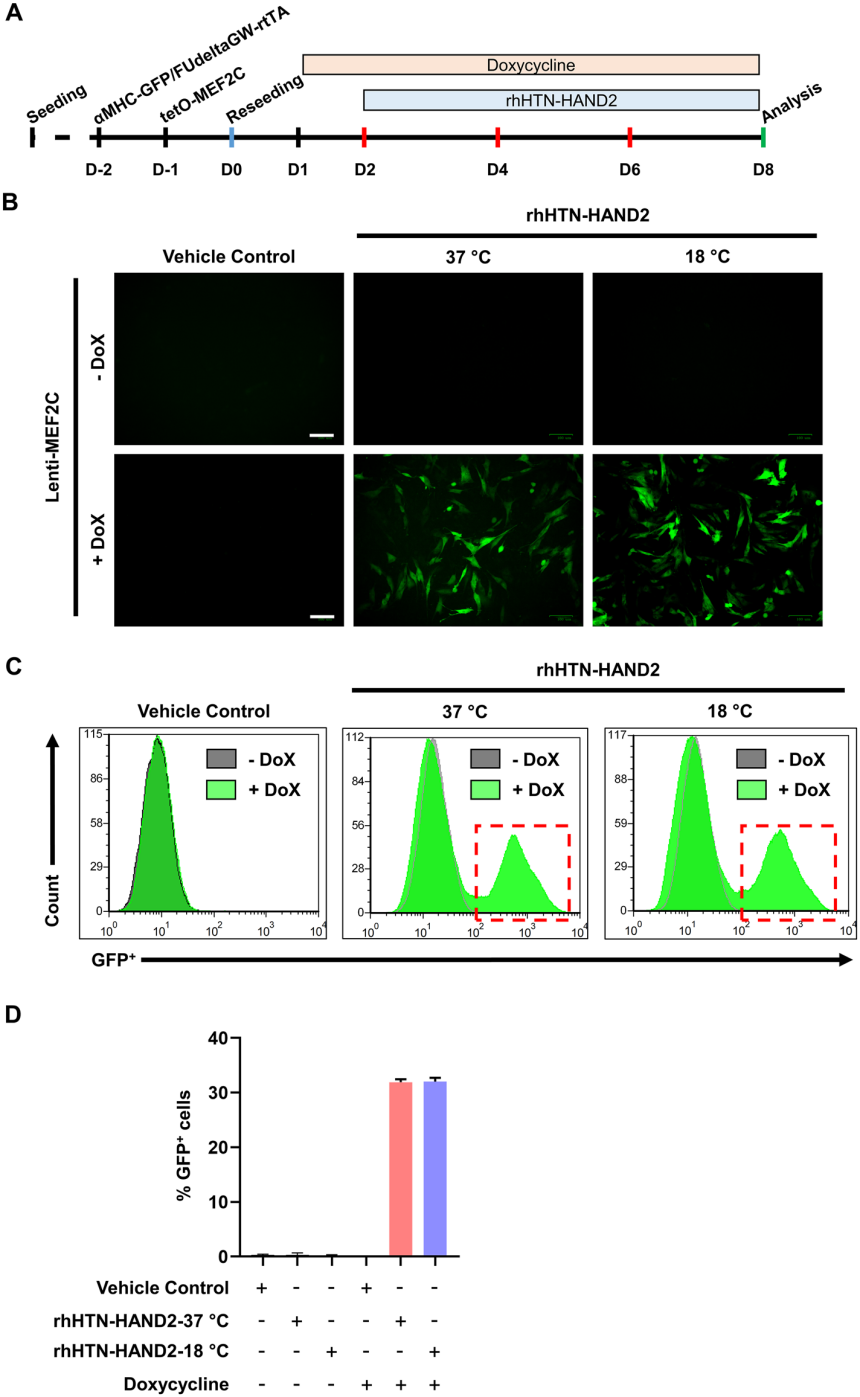
Effect of the purified rhHAND2 fusion protein on synergistic activation of the α-MHC promoter in the presence of MEF2C. (**A**) Schematic representative of the strategy employed to analyze the transcriptional activity of the purified rhHTN-HAND2 protein. (**B**) Firstly, H9C2 cells were transduced with α-MHC-eGFP, FUdeltaGW-rtTA, and tetO-MEF2C viral vectors. Subsequently, they were treated with doxycycline and purified rhHTN-HAND2 protein (induced either at 37 or 18 °C) or vehicle control. After 8 days, images were captured and analyzed. All images were taken with identical camera settings. Scale bar: 100 μm. (**C**) Flow cytometry analysis of GFP^+^ cells. (**D**) Quantitative representation of (**C**). The quantitative data shown are mean ± SEM (n = 4). Dox: doxycycline.

To further examine the transcriptional activity of this rhHAND2 fusion protein, we selected one of its downstream targets, a cardiomyocyte-specific α-MHC gene, and performed the target promoter-driven eGFP reporter assay. It has been reported that HAND2, along with another cardiac restricted transcription factor, MEF2C, synergistically activates α-MHC and atrial natriuretic peptide (ANP) genes^60^. We first confirmed the exogenous expression of MEF2C only upon induction with doxycycline in H9C2 cells post-transduction with FUdeltaGW-rtTA and inducible tetO-MEF2C lentiviral vectors (Fig S5). For analyzing the synergistic activation of α-MHC, we transduced the H9C2 cells with lentiviral vectors followed by treatment with rhHTN-HAND2 protein along with doxycycline (Fig. 9A). Remarkably, the GFP^+^ cells were observed in both the purified fusion proteins (induced at 37 and 18 °C) treated samples (Fig. 9B). In fact, expression of GFP was observed as early as day 4 due to the transcriptional activation of its α-MHC promoter by rhHTN-HAND2 and MEF2C proteins. Flow cytometry analyses showed that around 32% of the population were GFP^+^, whereas < 1% of GFP^+^ cells were observed in the other conditions (Fig. 9C,D). Our results thus signify that along with MEF2C, our purified rhHAND2 fusion protein, independent of the induction temperature, synergistically activated and induced the expression of α-MHC promoter-driven eGFP, thereby confirming its biological activity.

## Discussion

Here we demonstrate the purification of a transducible version of the bioactive rhHAND2 fusion protein, a crucial cardiac reprogramming factor to derive functional myocytes from the non-myocytes. Several studies have reported that the inclusion of this HAND2 transcription factor in GATA4, MEF2C, and TBX5 cocktails improved the cardiac reprogramming efficiency^26–28^. All these studies used the genetic form of this reprogramming factor and delivered it through viral vectors to derive induced cardiomyocytes, thereby limiting its therapeutic applications. On this account, generating a transducible and biologically active HAND2 protein is crucial to obviate the potential risks associated with using viruses and DNA transfection in cardiac reprogramming. An effective alternative to an integrative approach is the incorporation of recombinant proteins into the mammalian cells, as this approach is devoid of unnecessary integration and genomic alterations^13–17^.

First and foremost, we codon-optimized the *HAND2* protein-coding sequence to express it in the heterologous system efficiently. Several studies on multiple genes have demonstrated the enhanced heterologous expression of human genes in *E. coli* after codon optimization^50,52,61^, eliminating the roadblocks associated with the protein expression, namely, codon bias, destabilizing RNA elements, non-optimal GC content, and so forth. To overcome these roadblocks, we have codon-optimized the *HAND2* gene for its expression in *E. coli*. Consistent with our previous studies^30–33^; here also we found an improvement in the codon adaptation index of codon-optimized *HAND2* sequence than its non-optimized sequence. Results further established that the *HAND2* sequence is devoid of any rare codons after codon optimization.

The expression host used in this study is *E. coli*. It has a high transformation efficiency and is economical for recombinant protein production in large quantities, fast growth, and well-known genetics^61,62^. In addition, this organism is commonly used to produce human recombinant proteins that do not require posttranslational modifications for functionality^33,39,42,43,63–68^. Phosphorylation of HAND2 by Akt leads to the formation of the heterodimer, thereby inhibiting its transcriptional activity^69^. To circumvent this posttranslational modification, particularly *E. coli* strain BL21(DE3) (lacks *lon* and *OmpT* proteases) was used for the stable expression of rhHAND2 fusion proteins. This strain is enhanced with T7 RNA polymerase, compatible with the pET28a(+) expression vector, and ideal for the overexpression and stability of the desired protein^70^.

In the present study, we report for the first-time screening and identification of the ideal expression parameters for the soluble expression of rhHAND2 fusion proteins in *E. coli*. Consistent with this, we and others have previously demonstrated the effect of expression parameters on the expression, solubility, stability, and secondary structure conformation of recombinant proteins^30–33,39–50^. Moreover, we also observed the effect of fusion tags´ position on the expression and production of the quality rhHAND2 fusion protein, similar to our previous observations with ETS2, MESP1, and TBX5 recombinant proteins^30,31,33^. To the best of our knowledge, this is the first study to establish a one-step purification to obtain a highly pure rhHAND2 fusion protein under native (from soluble cell fraction) conditions that has its secondary structure retained. It can transduce into the mammalian cells and translocate to its nucleus without a transduction reagent. Similar strategies have been reported earlier by numerous studies for recombinant transcription factors purified from *E. coli*, namely, OCT4, NANOG, SOX2, ETS2, PDX1, MESP1, GATA4, NGN3, GLIS1, and TBX5^13,30–33,37,39–43,71,72^. Our strategy of coupling this transcription factor with the protein transduction domain and NLS facilitated its delivery into the mammalian cell target site. As a transcription factor, it has a major role inside the nucleus; therefore, its nuclear entry is one of the most critical aspects of its functionality. Similar fusion strategies were employed in previous studies, including ours, for the efficient subcellular and subnuclear delivery of reprogramming factors such as OCT4, NANOG, SOX2, GATA4, NGN3, and TBX5 in the form of recombinant proteins in mammalian cells^32,33,37,42,43,71–74^. These studies have used TAT and NLS as fusion tags to efficiently deliver protein of interest into the cell and its nucleus, respectively, to exert their biological function in mammalian cells^13,32,33,37,42,43,71,72^.

The generated transducible version of rhHAND2 fusion protein does not require any additional protein transduction reagent for its effective delivery to the target site in mammalian cells^13,75^. Previous studies have employed cell-permeant recombinant transcription factors in the cell reprogramming process and established that their functionality is comparable to their genetic forms^73,74^. Most importantly, in all these studies, the presence of the fusion tags (TAT and NLS) did not hinder their functionality. Hence, this purified HAND2 recombinant protein can be utilized to generate transgene-free cells as well as for other biological applications, thus, eliminating the limitations that accrue due to the plasmid or viral-based approaches^76,77^.

Since HAND2 has been reported to promote angiogenesis^23,59^, the effect of its pure recombinant fusion protein version in the neovascularization was investigated in this study. The results showed that the rhHTN-HAND2 protein promoted the migration of endothelial cells and induced neovascularization by sprouting angiogenesis. The endothelial cell migration and angiogenic potential of this purified fusion protein was comparable to the VEGF, a crucial inducer of angiogenesis. Similar observations of neovascularization were reported, induced by *E. coli-*derived recombinant asparaginyl-tRNA synthetase^35^ and dermatopontin^36^ proteins. It is reported that HAND2 regulates angiogenesis through the Notch signaling pathway^59^. Thus, the rhHAND2 fusion protein generated by us might also induce neovascularization through the Notch signaling pathway. Further detailed studies are required to confirm the same.

We also showed that the rhHTN-HAND2 protein regulates α-MHC synergistically with MEF2C. These observations were in line with the previous study, showing the synergistic activation of the α-MHC promoter by HAND2 and MEF2C^60^. Interestingly, studies reported the direct interaction of HAND2 with MEF2C in vitro and in vivo^60,78^. Thus, HAND2 and MEF2C interact to form a protein complex, which then regulates transcription. Physiologically, HAND2 has been identified in both homodimer and heterodimer forms. HAND2 forms heterodimers with the ubiquitously expressed protein (majorly bHLH proteins) to enhance its transcriptional activation^79,80^. Altogether, our results confirm that this purified rhHTN-HAND2 protein is biologically active irrespective of the induction temperature.

In summary, we have successfully conducted codon optimization, molecular cloning and expression, established one-step homogeneous purification of the full-length human HAND2 reprogramming factor from *E. coli* in the form of recombinant protein, and validated its transcriptional activity. The established approach is simple, cost-effective, and highly reproducible. We have identified the optimal expression conditions and showed the impact of the position of fusion tags on the rhHAND2 fusion protein expression. Our highly pure recombinant version of HAND2 protein had retained its folding conformation and confirmed its ability to transduce the cells as well as translocate to the nucleus. We have also demonstrated its migration-inducing and angiogenic potential using scratch wound healing assay and CAM assay, respectively. Moreover, induction of α-MHC expression by the rhHAND2 protein in H9C2 cells in which MEF2C is overexpressed proves that the purified protein is biologically active. Amongst the plethora of possible biological applications of the rhHAND2 fusion protein, it can also be used as a substitute against its viral and genetic form in a direct cardiac reprogramming process to investigate its mechanistic role.

## Supplementary Information

Supplementary Information.

## Data Availability

The codon-optimized nucleotide sequence of *HAND2* gene (for expression in *E. coli*) can be accessed via GenBank using the accession code MW570765.
