# Supplementary material for: Generation of a recombinant version of a biologically active cell-permeant human HAND2 transcription factor from E. coli
Source: Sci Rep. 2022 Sep 27;12:16129. doi: 10.1038/s41598-022-19745-w (PMC9515176; doi:10.1038/s41598-022-19745-w)
Supplement: Supplementary file 1 — Supplementary Information. [file 41598_2022_19745_MOESM1_ESM.docx]

*Supplemental information*

**Generation of a recombinant version of a biologically active cell-permeant human HAND2 transcription factor from**

***E. coli***

**Krishna Kumar Haridhasapavalan^1^,** **Pradeep Kumar Sundaravadivelu^1^,** **Neha Joshi^2^, Nayan Jyoti Das^1^, Anshuman Mohapatra^3^, Udayashree Voorkara^4^, Vishwas Kaveeshwar^5,^*, Rajkumar P Thummer^1^***

**^1^** Laboratory for Stem Cell Engineering and Regenerative Medicine, Department of Biosciences and Bioengineering, Indian Institute of Technology Guwahati, Guwahati – 781039, Assam, India.

**^2^** Organelle Biology and Cellular Ageing Lab, Department of Biosciences and Bioengineering, Indian Institute of Technology Guwahati, Guwahati – 781039, Assam, India.

**^3^** Department of Biosciences and Bioengineering, Indian Institute of Technology Guwahati, Guwahati – 781039, Assam, India.

**^4^** Department of Obstetrics and Gynaecology, SDM College of Medical Sciences and Hospital, Shri Dharmasthala Manjunatheshwara University, Dharwad – 580009, Karnataka, India.

**^5^** Central Research Laboratory, SDM College of Medical Sciences and Hospital, Shri Dharmasthala Manjunatheshwara University, Dharwad – 580009, Karnataka, India.

**Extended Materials and methods**

**SDS-PAGE and immunoblotting**

The protein concentrations of the samples were estimated using the Bradford assay kit (Bio-Rad) with bovine serum albumin as a standard. Protein samples were solubilized with Laemmli buffer at 95 °C for 5 mins. and resolved on 12% polyacrylamide gel. Precision Plus Protein™ Dual Color (Bio-Rad) was used as the protein marker (broad range). For Coomassie staining, the resolved polyacrylamide gel was stained with Coomassie Brilliant Blue G-250 (Merck Millipore) and then destained using 50% methanol and 10% acetic acid (Merck Millipore). For immunoblotting analysis, the resolved proteins from the polyacrylamide gel were transferred onto a nitrocellulose membrane (Bio-Rad) using a Pierce Power Blotter XL System (Thermo Scientific™, Bremen, Germany). After transfer, the membrane was blocked [with 5% nonfat milk in Tris-buffered saline (TBS) (HiMedia) with 0.1% Tween-20 (TBST) (Invitrogen)], and then incubated with primary followed by respective secondary antibodies (Table S5). Immunoblot was developed in the presence of a chemiluminescence substrate (Bio-Rad), and the image was recorded using a ChemiDoc™ XRS+ molecular imager equipped with Image Lab™ software (Bio-Rad).

**Far-ultraviolet circular dichroism spectroscopy**

The purified rhHAND2 fusion proteins were desalted and buffer exchanged using PD10 columns (GE Healthcare) against sterile 20 mM sodium phosphate buffer (pH 8; HiMedia). After desalting, the proteins were diluted with 20 mM sodium phosphate buffer to the final concentration of ~1.5 µM and then scanned using a J-1500 spectropolarimeter (Jasco) equipped with a thermoelectric cooling-based temperature control unit in a 0.1 cm path length quartz cuvette. Scanning was recorded as an average of five accumulations from wavelength 260 to 190 nm at a rate of 100 nm/min. with a data integration time of 1 s under continuous purging of nitrogen gas at a rate of 8.3 × 10^–3^ l/s at 25 °C. The Beta Structure Selection (BeStSel) *in silico* online tool was used to estimate the secondary structure content of the protein from their final CD spectrum.

**Immunocytochemistry**

Cells were washed twice with phosphate buffer saline, then fixed with 4% paraformaldehyde for 10 min. at room temperature, permeabilized with 0.1% Triton™ X-100 (Sigma-Aldrich) for 15 min. at room temperature and blocked with 0.5% bovine serum albumin (HiMedia), 0.15% glycine (Bio-Rad), and 10% normal goat serum (HiMedia) for 1 h at room temperature. After blocking, cells were stained with primary antibodies overnight at 4 °C followed by detection with respective secondary antibodies for 1 h at room temperature (Table S5).

**Supplementary Figure S1.** Codon optimization and cloning workflow.


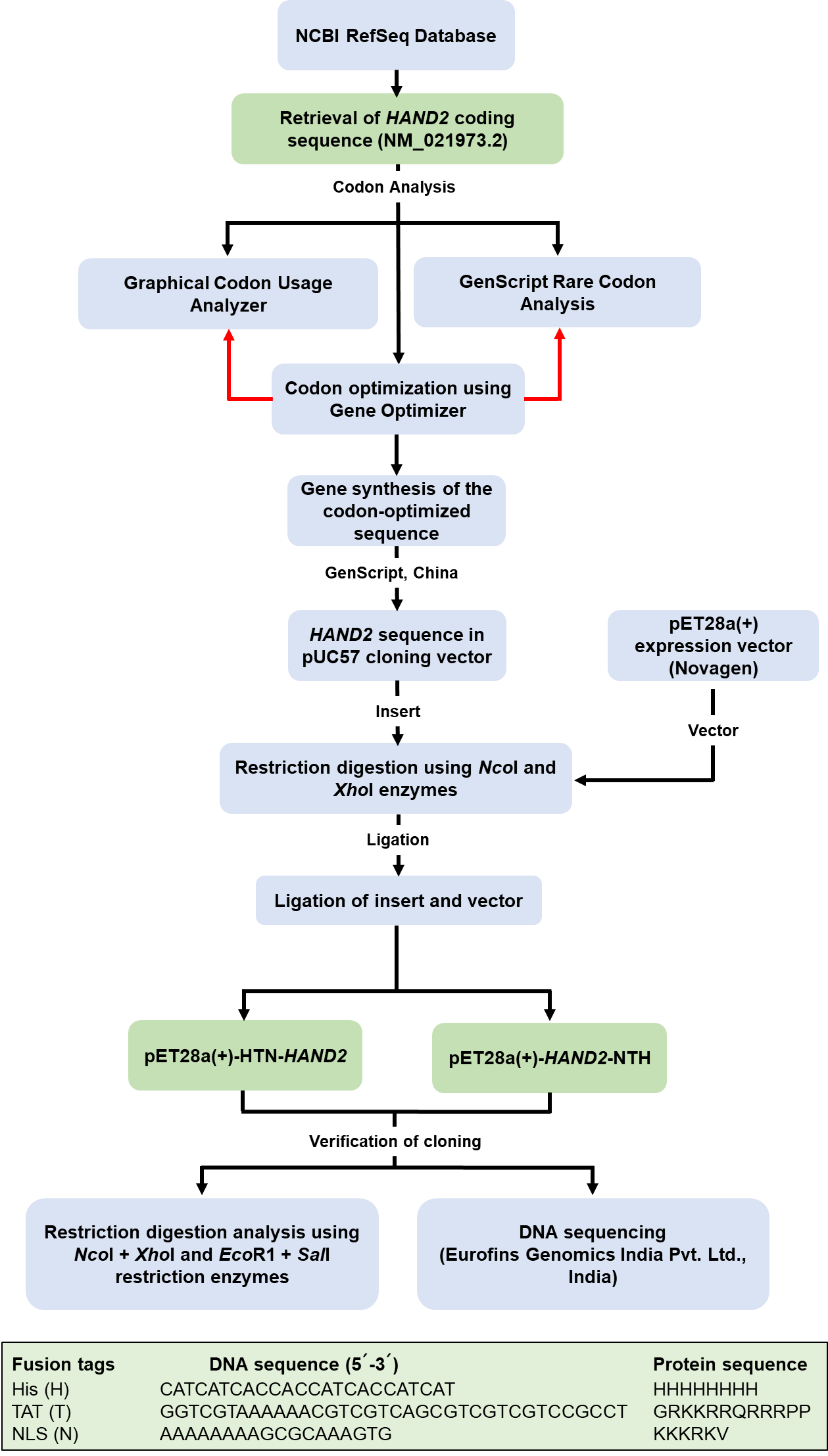


**Supplementary Figure S2.** Comparison of *HAND2* gene sequences before and after codon optimization. The codons before and after optimization are aligned with the respective amino acids. The change in nucleotides is represented in red.


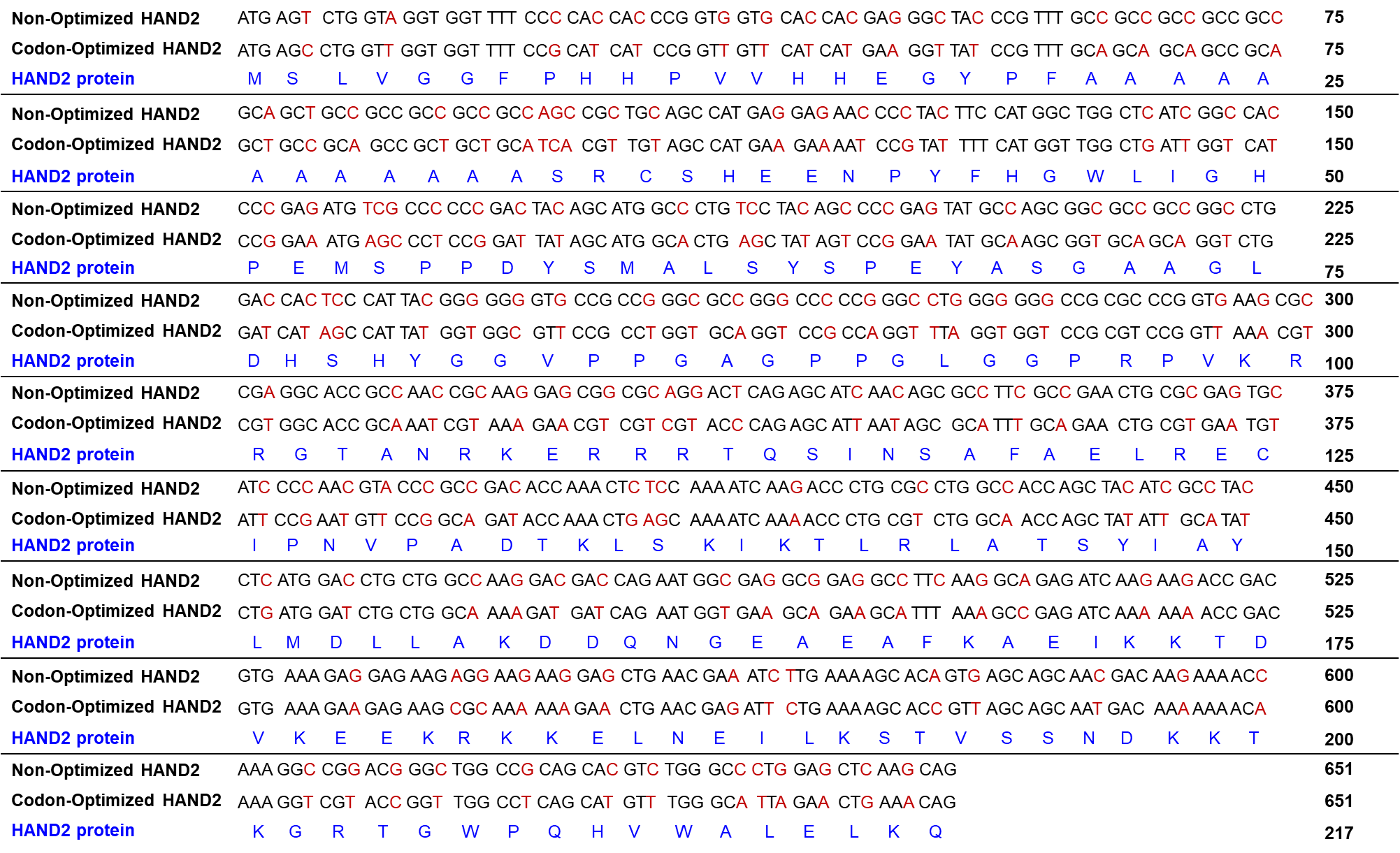


**Supplementary Figure S3.** Analysis of *HAND2* gene sequences before and after codon optimization using the GRCA online tool. The codons that are present in the 21-30 and below in the codon quality groups (a dotted line indicates the threshold) are rare codons and are more likely to diminish the expression of HAND2 in *E. coli*.


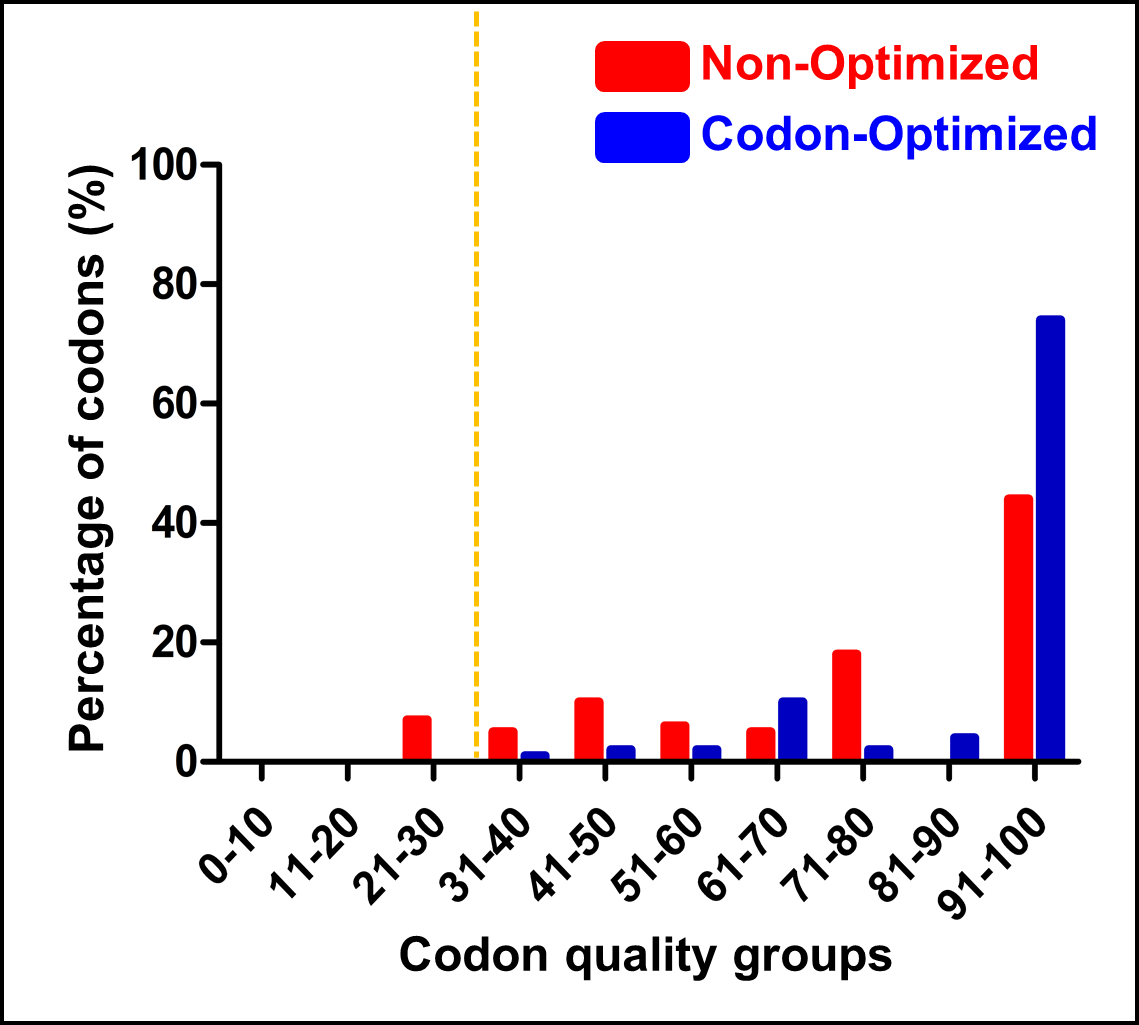


**Supplementary Figure S4.** Analysis of *HAND2* gene sequences before and after codon optimization using the GCUA tool. Codons with a relative adaptiveness value ≤ 30% (magenta) are likely to diminish the expression of HAND2 in *E. coli*.


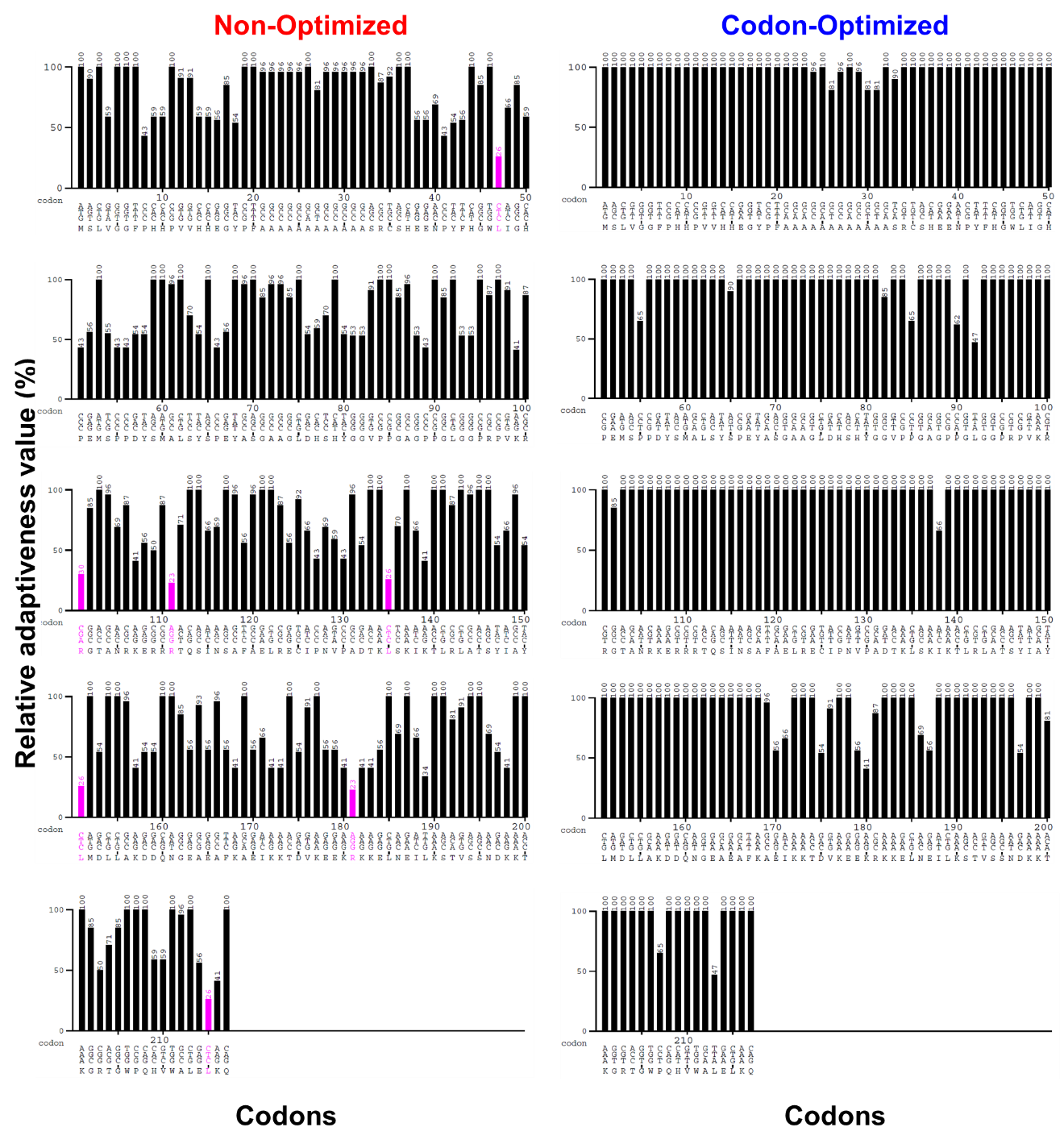


**Supplementary Figure S5.** Doxycycline-induced expression analysis of tetO-*MEF2C* transduced H9C2 cells. H9C2 cells were transduced with FUdeltaGW-rtTA and tetO-*MEF2C* lentiviral vectors in the presence of 5 µg/mL polybrene. On the next day, cells were washed with PBS and then induced with 2 µg/mL of doxycycline. Media was renewed every alternative day. After 4-5 days of induction, cells were harvested and lysed with RIPA lysis buffer (HiMedia). Samples were resolved on SDS-PAGE and then analyzed using immunoblotting with anti-Mef2c and anti-β-actin antibodies. M: protein marker.


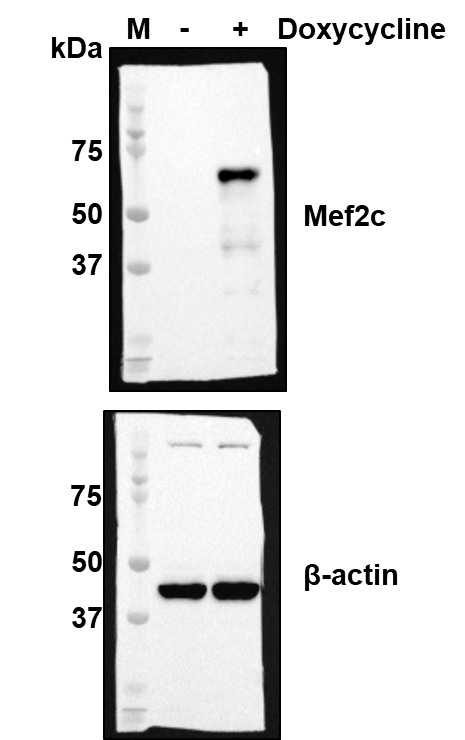


**Supplementary Figure S6.** Verification of the cloned plasmid pET28a(+)-HTN-*HAND2* using restriction digestion analysis. This figure is the uncropped image of Figure 1A (*bottom*).

**
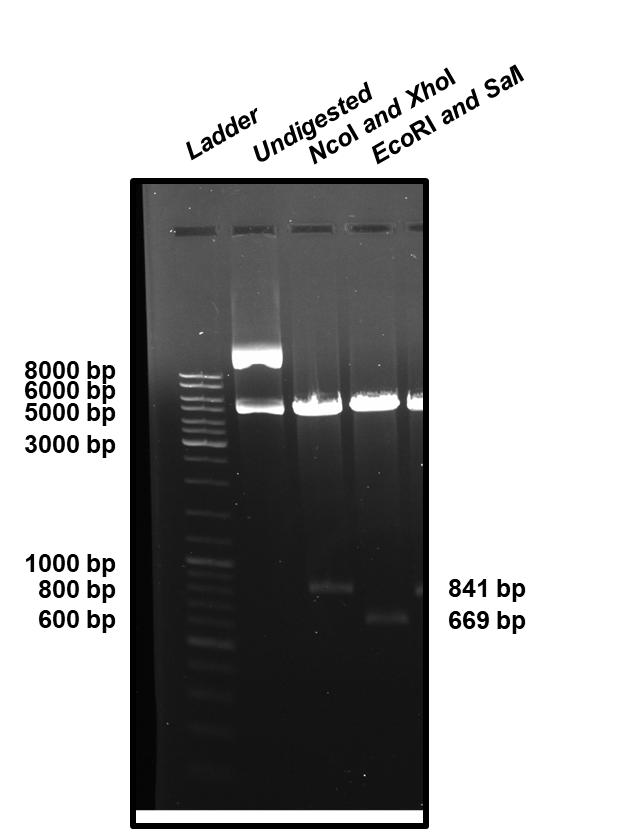
**

**Supplementary Figure S7.** Verification of the cloned plasmid pET28a(+)-*HAND2*­-NTH using restriction digestion analysis. This figure is the uncropped image of Figure 1B (*bottom*).

**
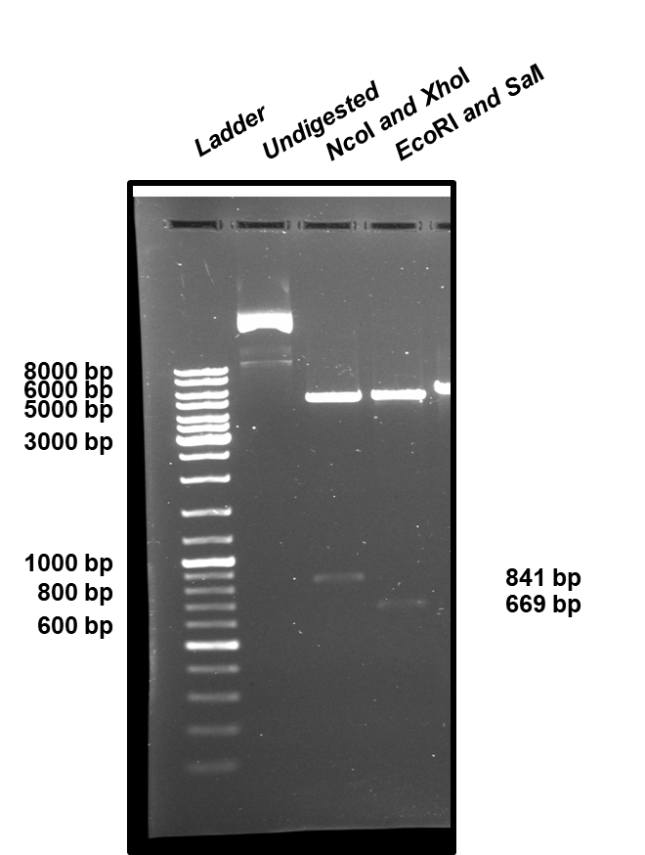
**

**Supplementary Figure S8.** The SDS-PAGE and immunoblotting analysis of the soluble expression of rhHTN-HAND2 protein when induced at two different temperatures. This figure is the uncropped images of Figure 2A. M: Protein marker (kDa); L: Total cell lysate; P: Pellet/insoluble cell fraction; S: Supernatant/soluble cell fraction.


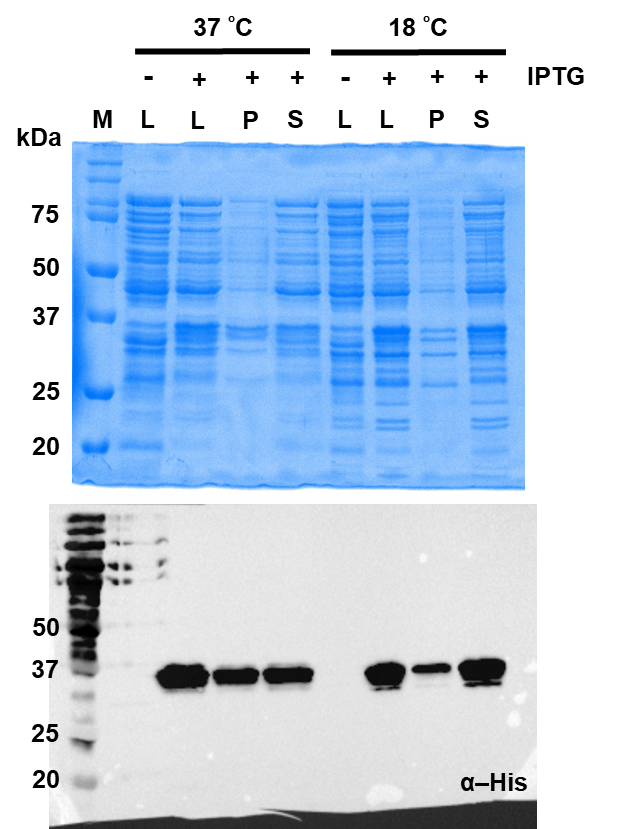


**Supplementary Figure S9.** The SDS-PAGE and immunoblotting analysis of the soluble expression of rhHAND2-NTH protein when induced at two different temperatures. This figure is the uncropped image of Figure 2B. M: Protein marker (kDa); L: Total cell lysate; P: Pellet/insoluble cell fraction; S: Supernatant/soluble cell fraction.


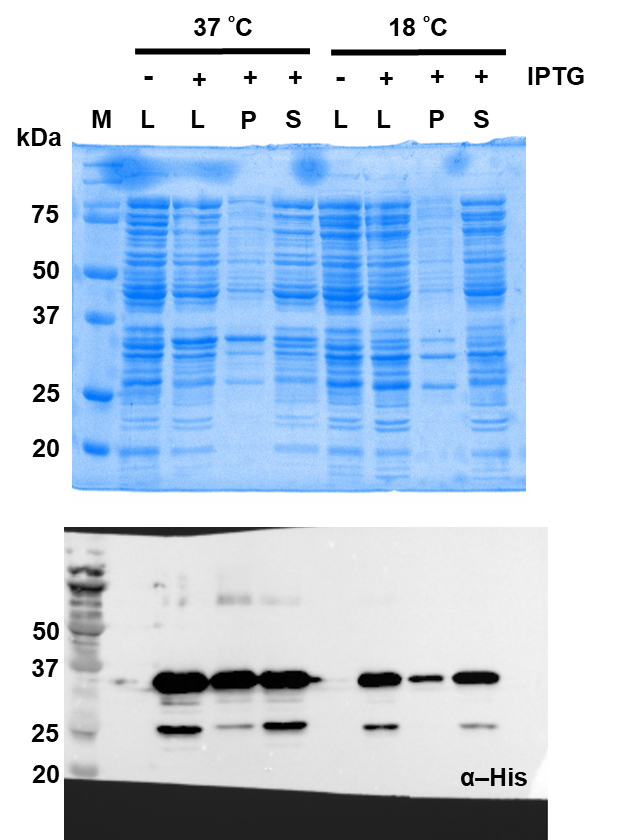


**Supplementary Figure S10.** The SDS-PAGE and immunoblotting analysis of the effect of imidazole concentration on the amount of HAND2 protein eluted. This figure is the uncropped image of Figure 3A. M: Protein marker (kDa).


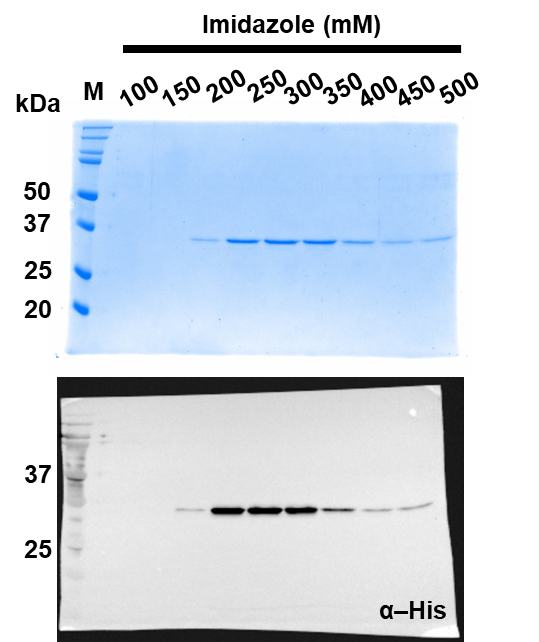


**Supplementary Figure S11.** The SDS-PAGE and immunoblotting analysis of the purification of rhHTN-HAND2 protein-induced at 37 °C. This figure is the uncropped image of Figure 4B. M: protein marker, L: lysate, S: soluble supernatant, F: flow-through, W1: wash buffer 1, W2: wash buffer 2, W3: wash buffer 3, E: elution.


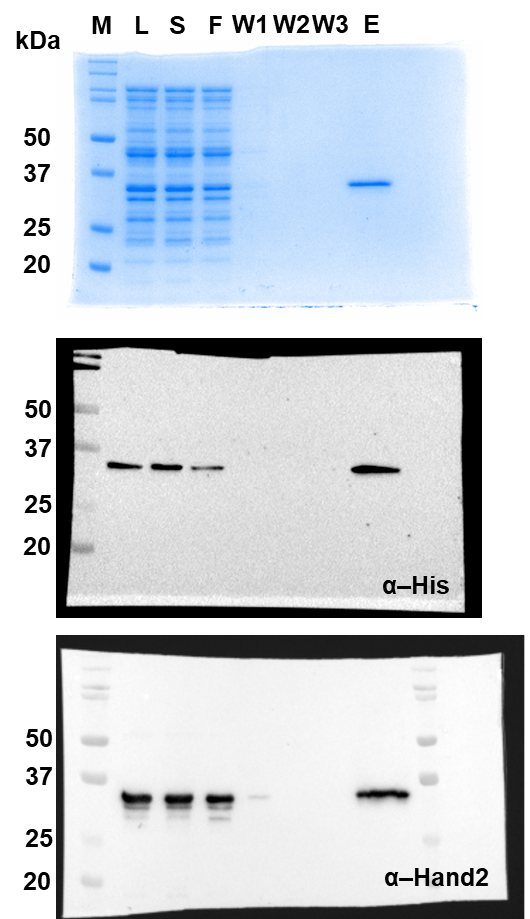


**Supplementary Figure S12.** The SDS-PAGE and immunoblotting analysis of the purification of rhHTN-HAND2 protein-induced at 18 °C. This figure is the uncropped image of Figure 4C. M: protein marker, L: lysate, S: soluble supernatant, F: flow-through, W1: wash buffer 1, W2: wash buffer 2, W3: wash buffer 3, E: elution.


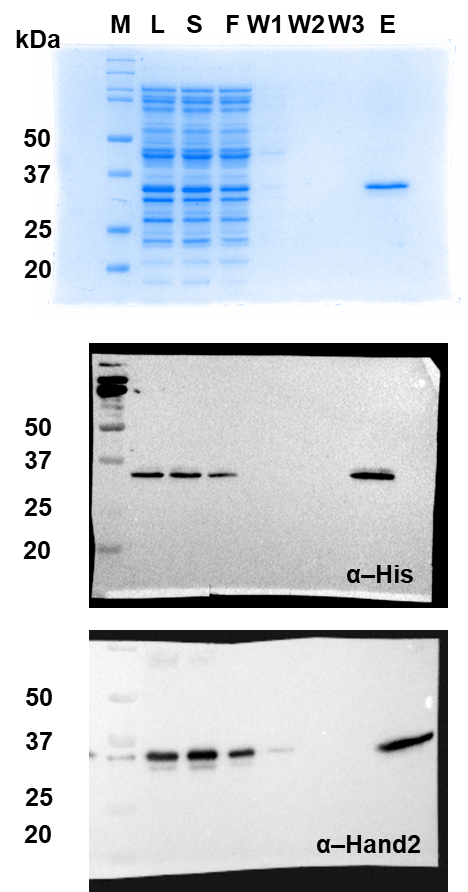


**Supplementary Figure S13.** The SDS-PAGE analysis of the elution profile of the purified rhHTN-HAND2 protein-induced at 37 °C. This figure is the uncropped image of Figure 4D. M: protein marker.


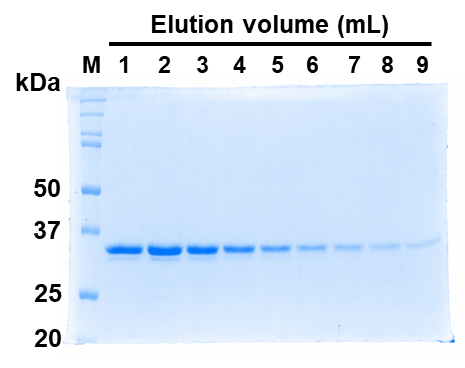


**Supplementary Figure S14.** The SDS-PAGE analysis of the elution profile of the purified rhHTN-HAND2 protein-induced at 18 °C. This figure is the uncropped image of Figure 4E. M: protein marker.


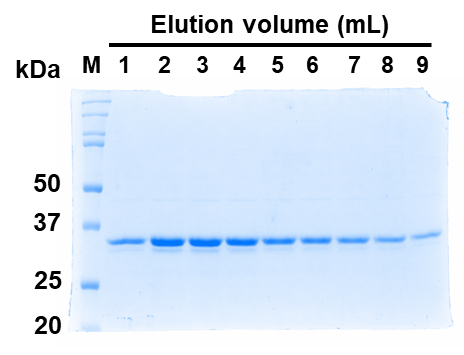


**Supplementary Table S1** List of *in silico* online tools used for codon optimization and evaluation

| **Online tools** | **Weblink** | **Reference(s)** |
| --- | --- | --- |
| **Codon optimization** | | |
| ThermoFisher Scientific GeneOptimizer | <https://www.thermofisher.com/in/en/home/life-science/cloning/gene-synthesis/geneart-gene-synthesis/geneoptimizer.html> | |
|  | | |
| **Evaluation of optimized codons** | | |
| Graphical Codon Usage Analyser 2.0 | <http://gcua.schoedl.de/> | |
| Genscript Rare Codon Analysis Tool | <https://www.genscript.com/tools/rare-codon-analysis> | |

**Supplementary Table S2** Primers used for verification of gene inserts by DNA sequencing

| **Primers** | **Sequence (5’ – 3’)** |
| --- | --- |
| Forward Primer | TAATACGACTCACTATAGGG |
| Reverse Primer | GCTAGTTATTGCTCAGCGG |

**Supplementary Table S3** Summarization of identified optimal expression parameters for the maximal soluble expression of rhHAND2 fusion protein in *E. coli*.

| Parameters | Values screened | Optimal value |
| --- | --- | --- |
| Pre-induction cell density (OD_600_) | 0.5, 1.0, and 1.5 | 0.5 |
| Inducer concentration (IPTG; mM) | 0.05, 0.1, 0.25, and 0.5 | 0.05 |
| Induction temperature (°C) | 37 and 18 | 37 and 18 |
| Induction incubation time (h) | For 37 °C: 2, 4, and 8  For 18 °C: 12, 24, and 48 | For 37 °C: 2  For 18 °C: 24 |

**Supplementary Table S4** Summary of purification buffers used and their composition

| Ingredients | Lysis buffer | Wash buffer | | | | | | Elution buffer | | Glycerol buffer | |  |
| --- | --- | --- | --- | --- | --- | --- | --- | --- | --- | --- | --- | --- |
|  |  | **W1** | | **W2** | | **W3** | |  |  |  |  |  |
| Phosphate buffer (mM) | 20 | | 20 | | 20 | | 20 | | 20 | | 20 | |
| NaCl (mM) | 150 | | 150 | | 150 | | 150 | | 150 | | - | |
| Imidazole (mM) | 20 | | 50 | | 100 | | 150 | | 300 | | - | |
| Glycerol (%) | 20 | | - | | - | | - | | 20 | | 20 | |

**Supplementary Table S5** List of antibodies used in this study

| Antibodies | Dilutions | | Source | Identifier |
| --- | --- | --- | --- | --- |
|  | Western blotting | Immunostaining |  |  |
| Anti-His | 1:5000 | N/A | BioBharati | Cat# BB-AB0010 |
| Anti-Hand2 | 1:2000 | 1:40 | Invitrogen | Cat#PA5-35186 |
| Anti-Mef2c | 1:2000 | N/A | Invitrogen | Cat#MA5-25477 |
| Anti-β-Actin | 1:5000 | N/A | BioBharati | Cat# BB-AB0024 |
| Anti-Rabbit IgG, HRP-conjugated | 1:5000 | N/A | Invitrogen | Cat#31460 |
| Anti-Mouse IgG, HRP-conjugated | 1:5000 | N/A | Invitrogen | Cat#31430 |
| Alexa Fluor 594 goat anti-rabbit IgG | N/A | 1:1000 | Invitrogen | Cat#A11037 |

N/A: not applicable

**Supplementary Table S6** Analysis of non-optimized as well as codon-optimized coding sequence of human *HAND2* gene using GRCA *in silico* tool

| Parameters | Non-optimized | Codon-optimized | Ideal range |
| --- | --- | --- | --- |
| Codon Adaptation Index | 0.69 | 0.89 | 0.8-1.0 |
| GC Content | 67% | 49% | 30-70% |
| Codon Frequency Distribution* | 7% | 0% | <30% |

* The percentage of low frequency (<30%) rare codons based on the target host organism.

**Supplementary Table S7** *HAND2* fusion gene constructs and expected size after its restriction digestion

| Vector/inserts/constructs | Restriction Enzyme(s) | Expected Size (bp) |
| --- | --- | --- |
| pET28a(+) Empty Vector | - | 5369 |
| *HAND2* | - | 654 |
| HTN-*HAND2* / *HAND2*-NTH | - | 839 |
| pET28a(+)-HTN-*HAND2* / pET28(+)-*HAND2*-NTH | - | 6064 |
| pET28a(+)-HTN-*HAND2* / pET28(+)-*HAND2*-NTH | *Nco*I and *Xho*I | 5231 and 833 |
| pET28a(+)-HTN-*HAND2* / pET28(+)-*HAND2*-NTH | *Eco*RI and *Sal*I | 5407 and 657 |
